# Multiscale affinity maturation simulations to elicit broadly neutralizing antibodies against HIV

**DOI:** 10.1371/journal.pcbi.1009391

**Published:** 2022-04-20

**Authors:** Simone Conti, Victor Ovchinnikov, Jonathan G. Faris, Arup K. Chakraborty, Martin Karplus, Kayla G. Sprenger

**Affiliations:** 1 Department of Chemistry and Chemical Biology, Harvard, Cambridge, Massachusetts, United States of America; 2 Department of Chemical and Biological Engineering, University of Colorado Boulder, Boulder, Colorado, United States of America; 3 Institute for Medical Engineering and Science, Massachusetts Institute of Technology (MIT), Cambridge, Massachusetts, United States of America; 4 Department of Chemical Engineering, MIT, Cambridge, Massachusetts, United States of America; 5 Department of Physics, MIT, Cambridge, Massachusetts, United States of America; 6 Ragon Institute of MGH, MIT and Harvard, Cambridge, Massachusetts, United States of America; 7 Department of Chemistry, MIT, Cambridge, Massachusetts, United States of America; 8 Laboratoire de Chimie Biophysique, Institut de Science et d’Ingénierie Supramoléculaires, Université de Strasbourg, Strasbourg, France; Johns Hopkins University, UNITED STATES

## Abstract

The design of vaccines against highly mutable pathogens, such as HIV and influenza, requires a detailed understanding of how the adaptive immune system responds to encountering multiple variant antigens (Ags). Here, we describe a multiscale model of B cell receptor (BCR) affinity maturation that employs actual BCR nucleotide sequences and treats BCR/Ag interactions in atomistic detail. We apply the model to simulate the maturation of a broadly neutralizing Ab (bnAb) against HIV. Starting from a germline precursor sequence of the VRC01 anti-HIV Ab, we simulate BCR evolution in response to different vaccination protocols and different Ags, which were previously designed by us. The simulation results provide qualitative guidelines for future vaccine design and reveal unique insights into bnAb evolution against the CD4 binding site of HIV. Our model makes possible direct comparisons of simulated BCR populations with results of deep sequencing data, which will be explored in future applications.

## Author summary

Vaccination has saved more lives than any other medical procedure. But, we do not have robust ways to develop vaccines against highly mutable pathogens. For example, there is no effective vaccine against HIV, and a universal vaccine against diverse strains of influenza is also not available. The development of immunization strategies to elicit antibodies that can neutralize diverse strains of highly mutable pathogens (so-called ‘broadly neutralizing antibodies’, or bnAbs) would enable the design of universal vaccines against such pathogens, as well as other viruses that may emerge in the future. In this paper, we present an agent-based model of affinity maturation–the Darwinian process by which antibodies evolve against a pathogen–that, for the first time, enables the *in silico* investigation of real germline nucleotide sequences of antibodies known to evolve into potent bnAbs, evolving against real amino acid sequences of HIV-based vaccine-candidate proteins. Our results provide new insights into bnAb evolution against HIV, and can be used to qualitatively guide the future design of vaccines against highly mutable pathogens.

## Introduction

Although great progress has been made in the development of therapeutics and annual vaccines against diseases such as HIV and influenza, respectively, the need for universal vaccines for these diseases remains high. Approximately 38 million people are still living with HIV today [1], and up to 650,000 annual deaths globally are still attributed to influenza [2]. Unfortunately, such viruses undergo rapid mutations that can render the host immune response ineffective, greatly reducing vaccine efficacy. Special classes of antibodies (Abs) called “broadly neutralizing antibodies”, or bnAbs, have now been discovered that are effective against HIV [3] and influenza [4]. However, it remains unclear how to elicit them via vaccination. This is largely due to an incomplete understanding of how the immune system responds to encountering multiple variant Ags, a situation that can arise during infection with a rapidly evolving pathogen.

B cell receptors (BCRs, which will eventually be secreted as Abs) evolve through the evolutionary process of affinity maturation (AM) [5], which occurs within Germinal Centers (GCs). The large diversity of the human germline BCR repertoire implies that, after encountering an Ag, some BCRs will bind to the Ag, albeit weakly. If the binding affinity is above a certain threshold, those B cells will get activated, undergo replication inside the GC, and accumulate mutations in their BCRs. The resultant mutated B cells are continuously recycled and selected on the basis of their affinity for the Ag [5].

In the presence of multiple Ags, which can be administered simultaneously or sequentially in a vaccine or be encountered naturally as mutated versions of the original infecting virus, B cells evolve to recognize each of the Ags to some extent. If the Ag binding sites (epitopes) presented to the immune system are sufficiently dissimilar, the activated memory B cells will experience conflicting selection forces, which have been described by a “frustration” parameter in our past work [6–8]. Changing the degree of dissimilarity between the Ags administered in a vaccine has been used to modulate the level of frustration imposed on GC reactions, and can have the effect of focusing BCR responses on conserved Ag residues, leading to the elicitation of bnAbs [6–8].

Past computational studies have identified additional variables that influence the evolution of BCRs, including Ag concentration [6,8] and the temporal pattern of Ag administration [7,8]. However, it remains unclear how to tune these variables to optimize bnAb production. Additionally, due to the coarse-graining used in past computational models of AM [6–15], most of these models cannot make predictions about the actual BCR and Ag sequences that will best promote bnAb formation during AM.

Several past computational studies have employed approximate AM schemes, in which Ab structures are used in protein docking, undergo multiple rounds of *in silico* mutagenesis, and have their binding free energy changes evaluated using force field-based scoring functions [16–18]. These studies use nucleotide sequences to represent the BCRs, but do not explicitly include important aspects of AM such as clonal competition and interference. Additionally, mutations are introduced into the BCRs on an *ad hoc* basis, neglecting the mutational biases of activation-induced cytidine deaminase (AID [19]), the enzyme that induces mutations during AM. Therefore, such approaches are unlikely to serve as reliable guides for studying the effects of vaccines on BCR/Ab evolution.

In this study, we describe a computational model of AM that (1) utilizes actual nucleotide sequences of BCRs and Ags, (2) employs an experimentally-informed model [19] for the identity and location of BCR nucleotide mutations introduced during AM, and (3) incorporates an efficient method to calculate BCR/Ag binding free energies. We believe this to be the most realistic model of AM currently available, which has the capacity to guide vaccine design efforts against highly mutable pathogens such as HIV and influenza.

As an application of the model, we study the evolution of the germline precursor of VRC01, a potent bnAb against the CD4 binding site (CD4bs) of HIV, in response to vaccination with multiple rationally-designed [20] HIV-based Ags administered in different temporal patterns. As in our past work [8], we assume that the desired germline B cells have already been activated [21]. Our results show that administering Ags in a temporal pattern that continuously increases the amount of imposed frustration maximizes the mean breadth of the resultant BCRs. We also find that administering certain Ags leads BCRs to evolve mutations that increase their degree of interfacial amino acid composition/electrostatic pattern matching against the CD4bs. We propose that this is an important mechanism by which maturing BCRs develop high affinity for the CD4bs of HIV.

## Results

### Simulated affinity maturation

**Fig 1:**
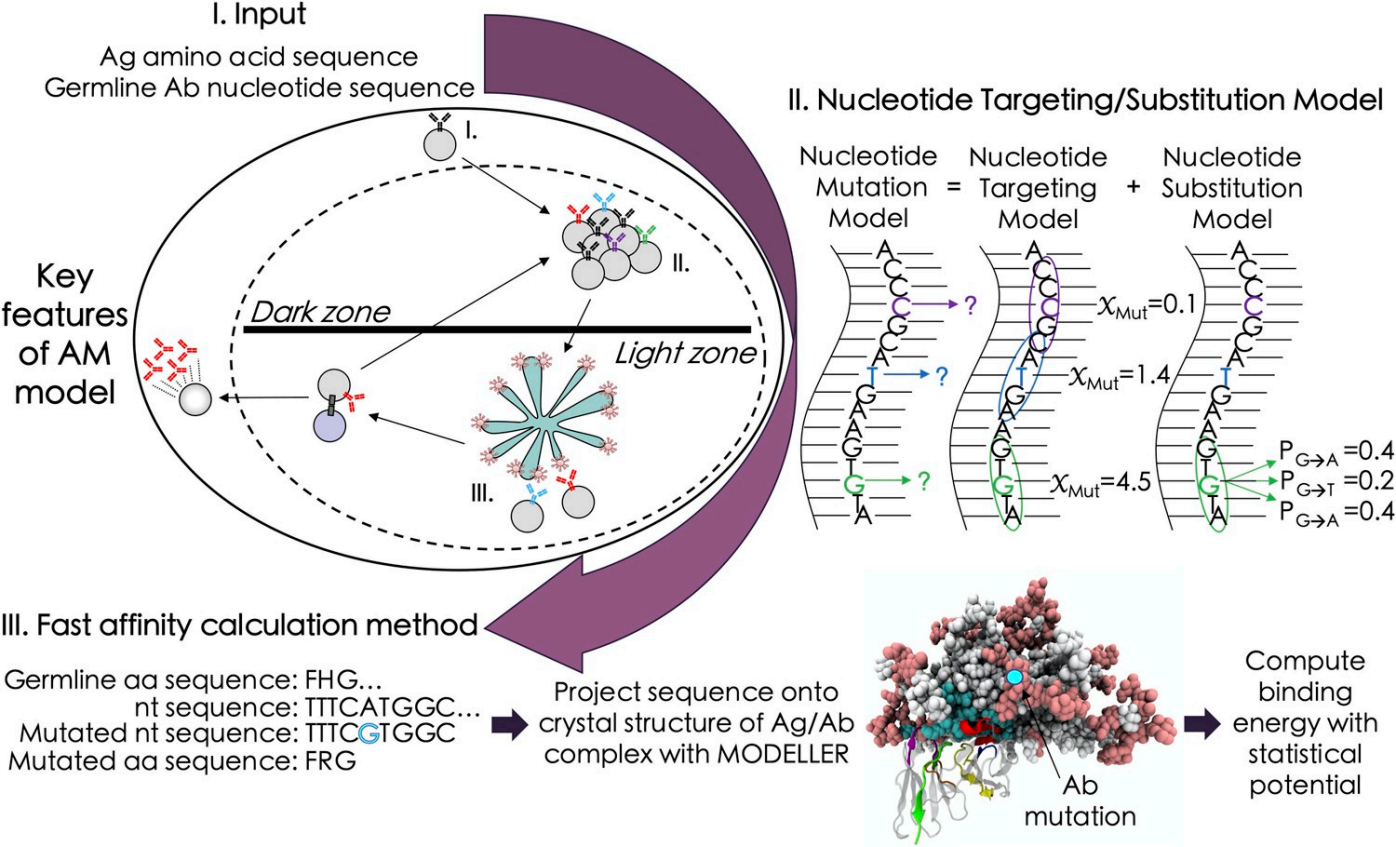
Simulation framework for the AM model, highlighting new features. Amino acid (aa) and nucleotide (nt) sequences of the Ag and germline BCR, respectively, are input into the model in step I. During B cell proliferation and mutation (step II), a mutation model [19] is used to determine where and which mutations will occur in the BCR sequences; nts that have a higher mutability score (*χ*_Mut_)–which accounts for the effects of surrounding nts–have a greater chance of being selected for mutation. This is followed in step III by BCR/Ag binding free energy calculations, which utilize the program Modeller [25] and published statistical potentials [26]. Final steps in the model include a second selection step, recycling, and differentiation (details in text). BCR breadth is computed as the fraction of Ags in a panel for which the BCR binds above a chosen threshold (see Methods).

A number of coarse-grained models to simulate AM are in the literature, many of which were developed within the past five years [6–15]. These models use coarse-grained representations for the BCR and Ag sequences, without a clear connection to the constituent nucleotides or corresponding amino acids. Here, we started from a coarse-grained model [8] and introduced actual nucleotide and amino acid representations for the BCRs and Ags, respectively. The model starts by seeding the GC with known germline BCR sequences (see Methods for details). We focus on HIV, and therefore seed the GC with the VRC01 germline [21] (VRC01GL) sequence, which is known to evolve into the potent bnAb VRC01. The founding B cell replicates without mutation or selection until the population reaches a size of 128 cells [22]. Then, the B cells start to accumulate mutations in their BCRs according to an AID-based mutation model [19], after which they compete for Ag and T cell help; 70% of the surviving B cells are selected randomly to undergo a new cycle of mutation and selection, and 30% exit as memory B cells [23]. The GC reaction ends when the B cell population recovers its initial size (successful outcome), or when all B cells die (unsuccessful outcome). In subsequent immunizations, the GC is seeded with three sequences randomly sampled from the memory BCR population produced during the previous immunization. An overview of the model is shown in Fig 1.

To reduce the computational complexity, we limited mutations to the complementarity-determining regions (CDRs) of the BCRs, though with a probability less than one (see Methods) to account for the fact that mutations can also occur in the framework (FRW) regions. We did not explicitly introduce framework mutations, since they are more likely to affect BCR stability than the BCR/Ag binding free energy, and would thus require additional complexity to accurately model [24]. Lastly, to compute the BCR/Ag binding free energy, we first create atomic models of the BCR/Ag complex with Modeller [25], and then evaluate the binding free energy using scoring functions from the literature [26]. On a standard 16-core CPU, each binding free energy calculation takes approximately one minute, rendering the AM simulations–which can require upwards of 15,000 calculations/GC–feasible. Overall, for the production runs and analysis carried out in this work, we computed nearly one million binding free energies for unique BCR/Ag complexes.

### Temporal pattern of Ag administration influences the breadth of the BCRs

We studied the outcomes of AM in response to 36 sequential vaccination protocols (Table A in S1 Text). The protocols are composed of a maximum of three sequential immunizations of four possible Ags, namely the wildtype BG505 SOSIP (WT) and three BG505-based derivatives designed to maximally promote bnAb evolution against the CD4bs of HIV [20]. These three designed Ags are identified as KR, EU, and HQ (see Methods). The concentration profile was kept the same across all vaccination protocols (see Methods); therefore, differences in simulation outcomes are due solely to differences in Ag sequences or temporal patterns of administration.

**Fig 2:**
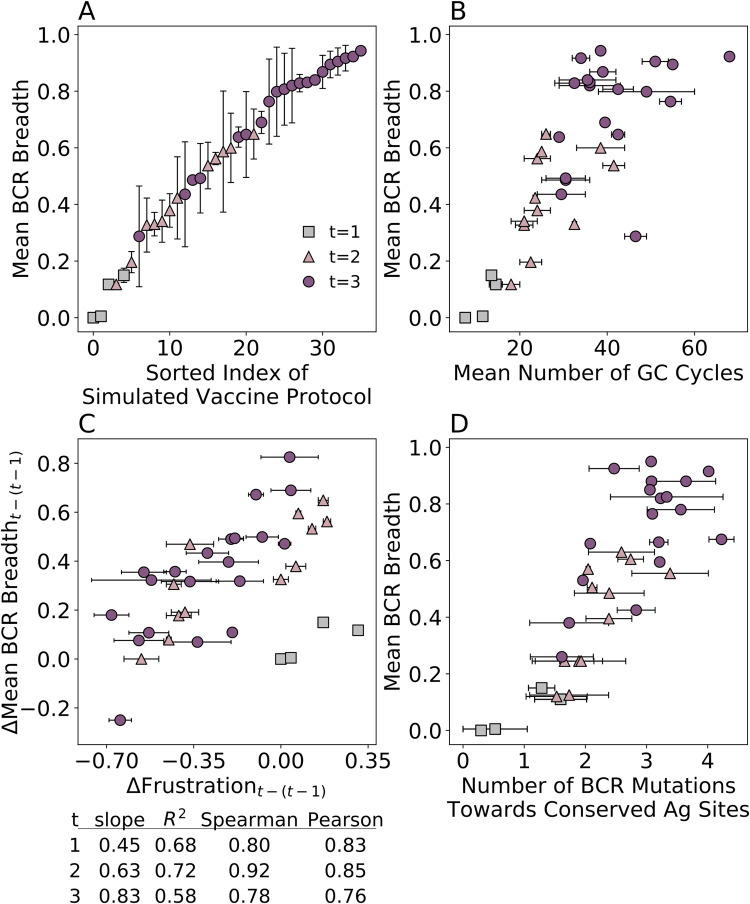
Results from AM simulations. (A) mean BCR breadth of vaccination protocols with t = 1, 2, and 3 single-Ag sequential immunizations, (B) mean BCR breadth vs. mean number of GC cycles, (C) changes in mean BCR breadth vs. changes in frustration between current (t) and former (t-1) immunizations, and (D) mean BCR breadth vs. mean number of mutations towards conserved Ag residues. In (C), linear fits were used to calculate the R^2^ values presented in the table below the graph. Error bars represent the standard deviation of two independent simulations of each vaccination protocol (except for the KR-KR-KR and HQ-EU-WT protocols, for which only one successful trial could be obtained), consisting of between one and three immunizations/GC reactions (error bars for mean BCR breadth are only shown in (A) for clarity).

Upon a single immunization with each of the four Ags, we find that the mean BCR breadth increases as follows: WT (breadth = 0.00) < KR (breadth = 0.005) < EU (breadth = 0.11) < HQ (breadth = 0.15). These results show that the choice of administered Ag has a substantial influence on breadth. We next investigated whether the mean BCR breadth could be optimized by administering these Ags in different temporal patterns (Fig 2A). Averaging the results across all 1-, 2-, or 3-Ag protocols, we find that the mean BCR breadth increases with the number of immunizations (breadth = 0.07, 0.42, and 0.74 for 1-, 2-, and 3-Ag protocols, respectively). However, the mean breadth is also observed to vary widely across the 2- or 3-Ag protocols, which shows that the temporal Ag administration pattern can have a large impact on BCR breadth. We also find that the mean number of GC cycles is correlated with breadth (Fig 2B), as more GC cycles afford BCRs more time to accumulate mutations that confer breadth.

A limitation of our model in its current form is the fact that administering sequential immunizations with the same Ag can result not only in a high degree of specificity for that Ag (which is expected), but also in a high mean BCR breadth (e.g., 0.90 for EU-EU-EU; see Table A in S1 Text). This observation–and the implication that affinity is correlated with breadth in our results–could stem from the fact that our model does not include some important factors known to constrain bnAb evolution *in vivo*. Such factors include glycans that shield conserved Ag residues [27] and mutations in variable Ag residues that can create loops to hinder access to conserved residues [28]. As a result, the probability of increasing affinity by mutating conserved versus variable residues is similar in our model, since the number of conserved and variable residues at the interface is similar. In our past work with a coarse-grained AM model [8], we addressed this issue by introducing a phenomenological parameter α that proportionally decreased the interaction strength between a BCR and a conserved Ag residue if the BCR made a mutation that strengthened interactions with a variable Ag residue (and vice versa). Future studies with the current model will hopefully capture these effects better as we build more complexity into the model and scoring function.

To understand how changing the temporal Ag administration pattern affects AM, we calculated the frustration imposed on the GC reactions by the different vaccination protocols, which was defined as the opposite sign of the average binding free energy of the GC-seeding BCR(s) and the Ag administered in the subsequent immunization. As such, the “frustration” is really a metric of the extent to which the existing B cell population is thrown off of a previous steady state by the introduction of a new Ag or change in concentration. The change in imposed frustration versus the change in mean BCR breadth between the current and former immunization is shown in Fig 2C. For 1-Ag protocols, frustration in the first immunization was compared to a reference immunization in which frustration was based on the binding free energy between VRC01GL and WT, the strongest binding Ag of the four. With this definition, the change in frustration is 0 for the 1-Ag protocol with WT (Fig 2C, blue square), and is greater than 0 for the other 1-Ag protocols. Since EU, KR, and HQ all bind less strongly to VRC01GL than WT, more B cells will die in response, even in the first immunization. The B cells that do survive fortunately make mutations that allow them to bind to both conserved and variable regions with a high affinity, affording them some small measure of breadth.

We find a positive correlation between the change in frustration and in the mean BCR breadth, with the increase being higher in each subsequent immunization (slopes and correlation data are reported in Fig 2C). In addition, with the current set of four Ags, we find that progressively fewer protocols result in actual increases in frustration (i.e., rather than smaller decreases in frustration; points to the right of zero on the x-axis) with each additional immunization. This could indicate that three sequential immunizations with these Ags are not necessary to elicit sufficiently high-breadth Abs. Mechanistically, this is because BCRs become increasingly capable of compensating for increasing frustration using strengthened interactions with conserved Ag residues. We demonstrated this by calculating the mean number of BCR mutations towards Ag residues considered to be conserved in the CD4bs [20] (see Methods, Table C in S1 Text). Fig 2D shows that the mean number of BCR mutations towards conserved Ag residues increases from 0.95 to 2.2 to 3.0 after one, two, and three immunizations, respectively. Consequently, we observe a strong positive correlation (R^2^ = 0.79) between the number of conserved site mutations and the mean BCR breadth.

In summary, we find that the elicitation of bnAbs is strongly dependent on the temporal pattern of Ag administration. Specifically, we find that vaccination protocols that impose the greatest increases in frustration from one immunization to the next result in the greatest increases in breadth. The increased frustration results in longer GC reaction times, which allow BCRs more time to acquire mutations that confer breadth. These results are consistent with our past studies with coarse-grained models. [8,29]

### BnAbs employ interfacial composition and electrostatic pattern matching (ICM) to bind to the CD4bs of HIV

**Fig 3:**
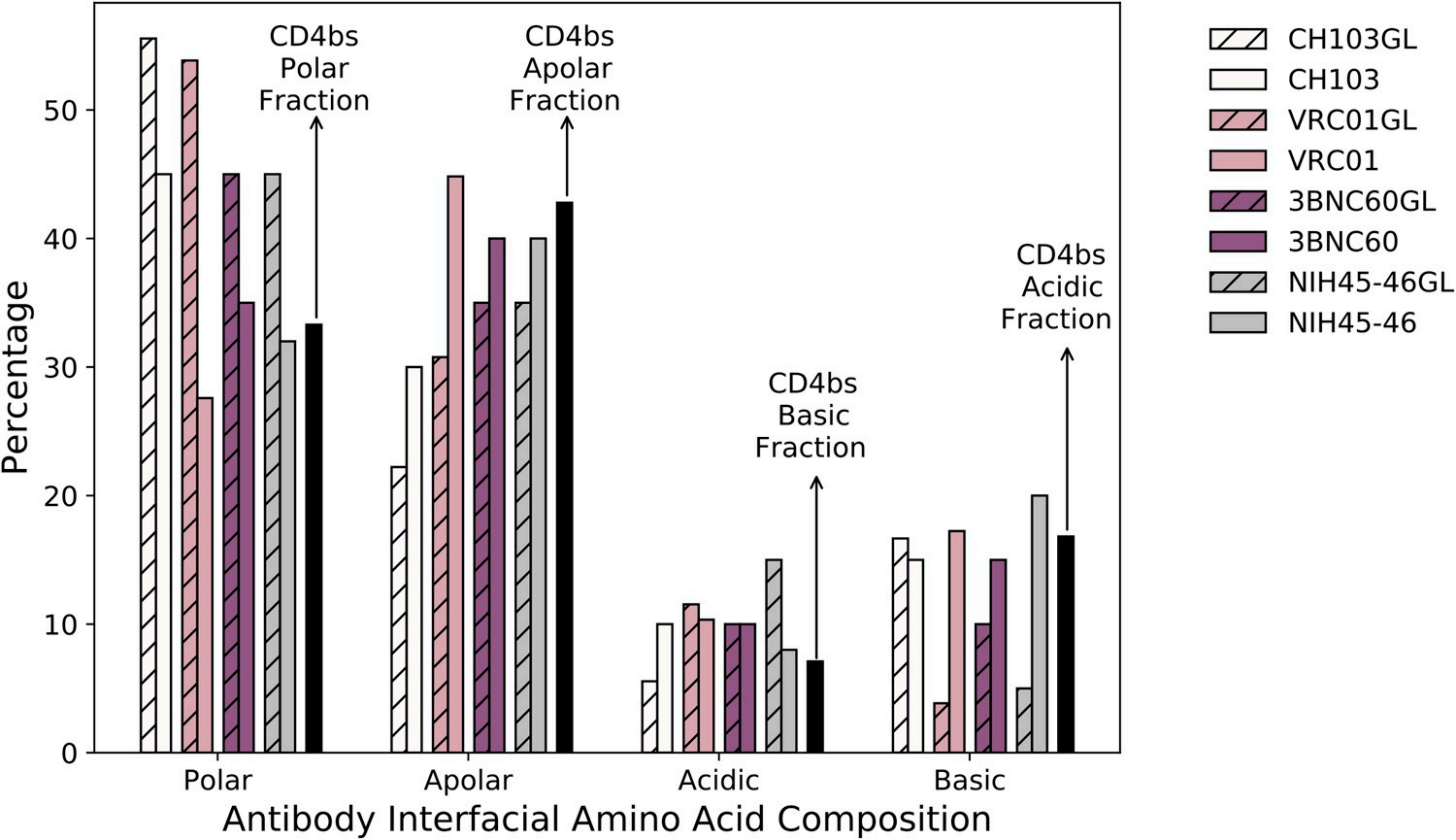
Anti-CD4bs bnAbs employ interfacial composition and electrostatic pattern matching (ICM) during AM. Interfacial amino acid compositions are shown in white (CH103), pink (VRC01), purple (3BNC60), and gray (NIH45-46), with solid and hatched bars representing the mature and germline forms of the Abs, respectively. For each amino acid type, the Abs’ target interfacial fraction in the CD4bs of HIV is indicated by a black bar (note that the Abs’ acidic interfacial compositions evolve to match the basic interfacial composition of the CD4bs, and vice versa).

To provide context for our simulation results, we performed a detailed analysis of the interfacial mutations that VRC01GL evolved *in vivo* during its maturation into its bnAb form, VRC01. Analyzing crystal structures of VRC01GL [21] and VRC01 [30] in complex with HIV-based Ags, we identified 26 and 29 interfacial Ab residues, respectively, to be within 4Å of the Ag. For both sets of residues, we determined the fraction of polar, apolar, acidic, and basic amino acids (Fig 3, pink bars; see Methods). For the Ag, the same coarse-graining procedure was carried out on the interfacial residues of 106 HIV Ag sequences from the Seaman panel [31]. The results were then averaged across the panel (Fig 3, black bars; see Methods), in order to account for the highly mutable nature of HIV.

Before AM, VRC01GL had much higher and lower fractions of polar and apolar interfacial residues than the CD4bs, respectively (Fig 3; pink vs. black bars). However, in the case of the affinity-matured Ab, VRC01, these two categories match much more closely to those of the CD4bs. Moreover, the basic interfacial fraction increases from 4% in VRC01GL to 17% in VRC01, to match the acidic fraction of the CD4bs at 17%, and the acidic interfacial fraction decreases from 12% in VRC01GL to 10% in VRC01, to better match the basic fraction of the CD4bs at 7%. Overall, a trend towards interfacial composition and electrostatic pattern matching (ICM) was observed between the evolving VRC01 Ab and the Ag binding site.

To validate these results, this analysis was performed on three other bnAbs targeting the CD4bs, for which both the germline and mature sequences were available: NIH45-46 [32,33], 3BNC60 [32], and CH103 [34] (see Methods). Fig 3 shows that these additional Abs also employed ICM during their maturation. This is particularly interesting for CH103, which binds to the CD4bs in a markedly different pose than the other bnAbs we considered (Fig A in S1 Text). Overall, these results imply that ICM is likely to be a general mechanism employed during AM and is an observable that can be monitored in the simulations. In the SI, we explore the biological driving forces behind these results, which include differences in the mutability of the codons encoding different amino acid types at the interface, and the number of nucleotide mutations required to transition from one amino acid type to another.

**Fig 4:**
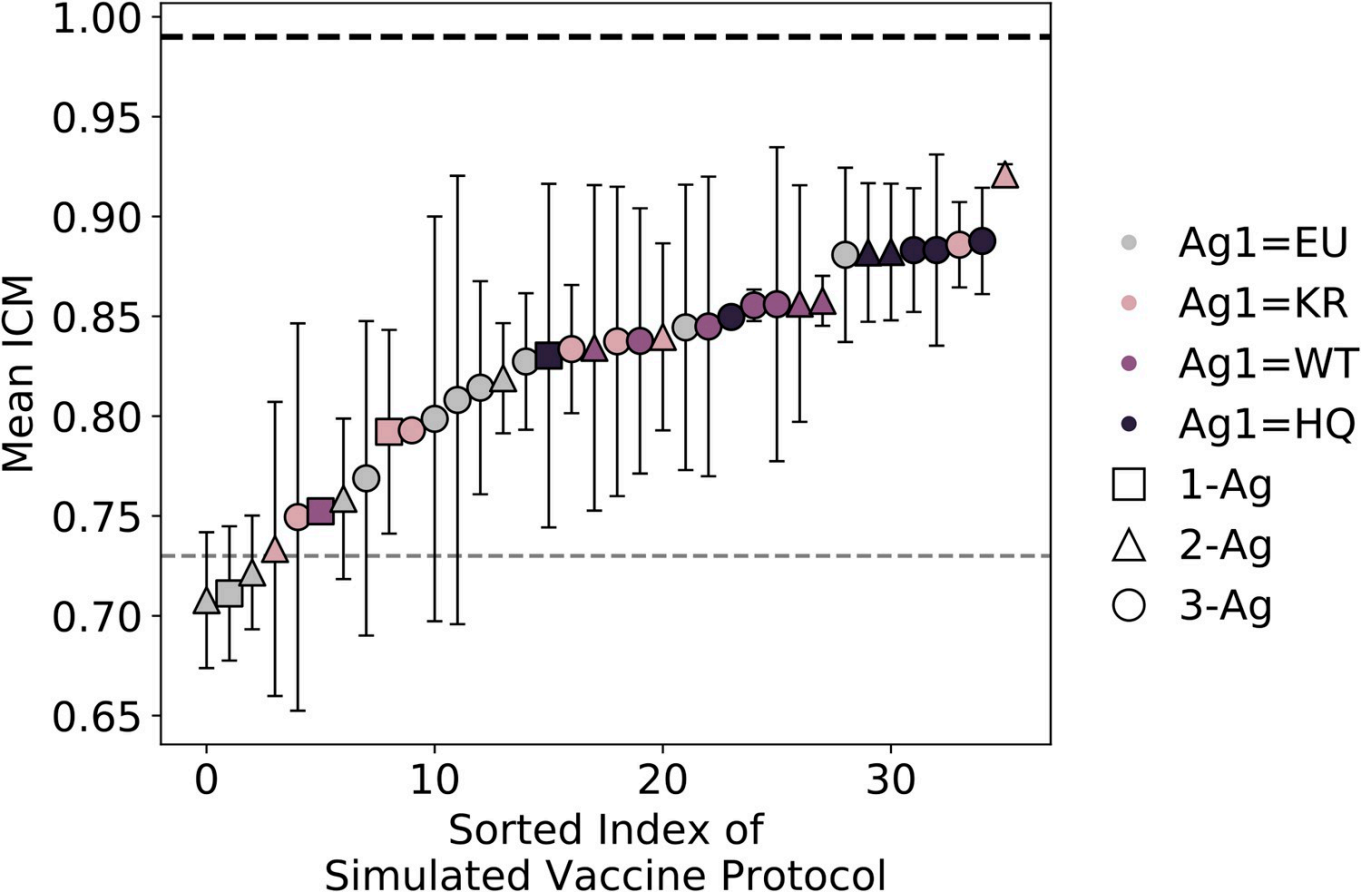
Results from AM simulations: mean weighted degree of interfacial composition and electrostatic pattern matching (ICM) of all 1-, 2-, or 3-Ag protocols, sorted by the mean ICM score. Error bars represent the standard deviation of two independent simulations of each vaccination protocol (except for the KR-KR-KR and HQ-EU-WT protocols, for which only one successful trial could be obtained), consisting of between one and three immunizations/GC reactions. Gray and black dotted lines indicate the respective values for VRC01GL and VRC01.

The degree of ICM of the *in silico-*produced BCRs was quantified using a chi-squared statistic on 22 interfacial residues in each BCR sequence (see Methods). The ICM score measures the summed deviation of the interfacial fraction of each amino acid type from its value in the CD4bs of HIV (n.b., the fraction of acidic residues is compared to the fraction of basic residues in the CD4bs, and vice versa; see Eqs 3, 4 in Methods), subtracted from unity. The ICM scores calculated for VRC01GL and VRC01 are 0.73 and 0.99, respectively. Computing the mean ICM score for the 36 vaccination protocols, we find that among the protocols with the same first administered Ag, the resultant BCRs tend to increase their ICM scores with each subsequent immunization (Fig 4 and Fig D in S1 Text), in line with the biological trends observed for the four bnAbs discussed above. Among protocols starting with each of the four Ags, those protocols in which the first administered Ag is EU, WT, or HQ consistently resulted in relatively low, intermediate, and high degrees of ICM, respectively. Protocols in which the first Ag was KR resulted in a wide range of ICM scores, implying that KR is overall a weak driver of ICM, despite the high average ICM score of the KR-KR protocol (0.92). Interestingly, we observe only a weak correlation between the degree of ICM and the mean breadth of the BCRs produced in the simulations (R^2^ = 0.22), though this correlation improves slightly upon reducing the stringency of the BCR breadth threshold (Fig C in S1 Text).

Given the low correlation observed between the degree of ICM and the mean BCR breadth, we sought to further explore the driving forces underlying ICM. To this end, we created a null model that introduces a mutation at the nucleotide level at uniformly-distributed random locations in the Ab sequence, rather than according to the previous 5-mer mutation model (see Methods for details). Using this null model, we performed two separate trials of three sequential immunizations with the WT Ag, keeping the concentrations of the immunizations (c1 = 0.24, c2 = 0.05, c3 = 0.01) as close as possible to those in the protocols performed using the 5-mer mutation model (c1 = 0.25, c2 = 0.05, c3 = 0.01). The results of these null model simulations are tabulated in Table A in S1 Text. The resulting mean BCR breadth is similar for the null model, producing values of 0.08, 0.36, and 0.59 for the WT, WT-WT, and WT-WT-WT protocols, respectively, compared to values of 0.00, 0.38, and 0.49 obtained using the 5-mer model. We also find the mean degree of ICM to be similar for the null model, producing values of 0.80, 0.84, and 0.92 for the WT, WT-WT, and WT-WT-WT protocols, respectively, compared to values of 0.75, 0.86, and 0.86 obtained using the 5-mer model. These results suggest that ICM increases primarily because of selection, rather than the skewed mutational distribution of the 5-mer model.

To understand why some Ags elicit BCR populations with higher ICM scores over others, we decomposed the ICM scores in Fig 4 into their individual components (i.e., changes in the interfacial fraction of each amino acid type in the BCR population over time; Fig D in S1 Text). Considering the results of Fig 4 and Fig D in S1 Text together, the results suggest trends exist between the types of BCR mutations made on a population level and the broad degree of ICM achieved by the BCR population. For example, Fig D in S1 Text, subplot A, shows protocols beginning with the EU Ag produce BCR populations that collectively mutate to decrease the fraction of acidic residues at the interface over time. Fig D in S1 Text, subplot A, also shows that some 1- and 2-Ag protocols starting with the EU Ag produce BCR populations that, on average, mutate to increase the polar interfacial fraction. Since these types of mutation result in interfacial BCR residue fractions that are further away from those of the CD4bs, these results provide a likely explanation for why EU-initiated protocols generally have low mean ICM scores (Fig 4). Conversely, Fig D in S1 Text, subplot D, shows protocols beginning with the HQ Ag produce BCR populations that collectively mutate to decrease the fraction of polar residues at the interface and increase the fraction of basic residues at the interface over time (note, these results do not imply polar-to-basic mutations occurred within the same BCR in a given round of AM). Since these types of mutation bring interfacial BCR compositions closer to those of the CD4bs, these results likely contribute to the high mean ICM scores of most HQ-initiated protocols (Fig 4). Similar analyses for protocols beginning with the KR and WT Ags are included in the SI.

## Discussion

Conventional vaccination approaches to evolve antibodies (Abs) against pathogens such as HIV and influenza are largely unsuccessful due to high mutability or antigenic drift, respectively. Broadly neutralizing Abs (bnAbs) could overcome this challenge, though it remains unclear how to elicit them via vaccination. Consequently, research focus is shifting towards identifying vaccine design variables that can facilitate bnAb evolution during affinity maturation (AM); e.g., sequences and concentrations of the administered antigens (Ags) [6,7,35–41]. Previous computational studies utilized coarse-grained representations for the B cell receptors (BCRs) and Ags. In this work, we developed a multiscale computational framework that models the AM of actual BCR sequences in response to vaccination with atomic-resolution Ags. This model enables future comparisons between simulations and experimental results from, e.g., deep sequencing of BCR repertoires.

Changing the Ags and their concentrations during vaccination disrupts the normal progression of AM by perturbing the memory BCR population, imposing what we termed “frustration”, as in our past work [6–8]. Here, we manipulated the level of frustration by administering four variant Ags, three of which were explicitly designed to improve the breadth of the elicited anti-HIV Abs [20], in 36 different temporal patterns. Our results show that increasing the imposed frustration increases the number of GC cycles during which BCRs can accumulate breadth-conferring mutations. Increasing frustration counteracts the fact that BCR interactions with conserved Ag sites are strengthened with each subsequent immunization, effectively lowering the frustration. This finding is qualitatively consistent with results from our previous work with coarse-grained models [8,29] that showed increasing the frustration upon the second immunization imposed a stronger selection force on activated memory B cells to evolve additional mutations towards conserved Ag residues.

The above comparison is only qualitative because many more simulations could be performed with the coarse-grained model due to the much lower computational cost, enabling conclusions about B cell survival rates. However, if trends from our past work hold true here as well, they would imply that rather than attempting to maximize the level of frustration in each immunization, there is an optimally increased level of frustration to impose (i.e., Ag sequence to use, in this case) in each immunization. Such an approach is predicted to simultaneously optimize the production of high-breadth Abs and B cell survival rates [8].

An important finding from this work, which was not possible to obtain with previous coarse-grained models, is that the VRC01 bnAb evolved mutations at the interface that enabled interfacial composition/electrostatic pattern matching against the CD4bs itself. We showed that three other anti-CD4bs bnAbs also employed this mechanism–which we term ICM–during their evolution. Results from the AM simulations show that the produced BCRs also increase their degree of ICM over time, though the final degree of ICM achieved by the BCRs appears to depend strongly on the first Ag that they encounter. After immunizing with the first Ag, B cells are selected for mutations that improve their binding affinity to that particular Ag. The effect of these mutations on the degree of ICM varies widely depending on the Ag, in part because of the many possible affinity-increasing mutations. The dependence of ICM on the first administered Ag is likely diminished for anti-HIV bnAbs due to the numerous strains of the virus they encounter over their many years of maturation.

We discuss various driving forces behind ICM in the SI. In addition, better ICM may enable BCRs to more closely approach an Ag, thus allowing contacts with the conserved residues of the Ag. This, in turn, may increase the selection force to evolve mutations that bind better to the conserved residues, thus conferring breadth. Such a mechanism may promote bnAb evolution even though ICM per se does not correlate highly with breadth. Simulations performed using a null model in which random mutations in the Ab sequence were distributed uniformly rather than according to an AID-based ‘5-mer’ mutation model suggest that selection is a key driver of ICM. Both the mean breadth and degree of ICM of the Abs obtained using the null model were comparable to those obtained using the 5-mer model, implying the constrained mutational landscape resulting from AID biases is not very likely to be a determining factor in bnAb evolution.

Also, it remains unknown whether potent (but not necessarily broad) antibodies also increase their degree of ICM during maturation. This would indicate that ICM is a more general feature of antibody evolution, a pattern matching mechanism which allows for general compatibility in the interactions between BCRs and Ags, before precise mutations are made to enable BCR binding to conserved Ag residues. Future longitudinal studies of bnAb evolution may help to shed light on this factor.

Beyond the potential indirect effects of ICM on BCR breadth, many other factors are known to influence a BCR’s ability to bind to conserved Ag residues and acquire breadth, which were also mentioned earlier. These include geometric constraints that make it sterically difficult for BCRs to access conserved Ag sites (e.g., a high surface density of influenza’s hemagglutinin spike protein hinders BCR binding to conserved hemagglutinin stem epitopes [42]). Another example is the presence of glycans in the Ag structure that can shield conserved residues from BCRs, such as the N276 glycan that is located near the CD4bs of HIV and serves as a major barrier to bnAb evolution [27]. Lastly, mutations in variable Ag residues can also insert loops that further hinder BCRs’ abilities to bind to conserved residues and acquire breadth [28]. We plan to explore the effects of these factors on bnAb evolution in the future by building such complexities into our model.

In view of our results, we suggest that a promising route to a universal vaccine for HIV is to first administer an Ag that promotes BCR mutations which increase the degree of ICM against the CD4bs (although we acknowledge that designing such immunogens may be nontrivial). This step should be followed by sequential immunizations with Ags that have an optimal number of increasingly different amino acids in the variable residues surrounding the conserved residues to achieve an optimally increasing temporal frustration profile. This procedure is expected to optimize the breadth of the mature BCR/Ab population, and according to our past work [8], it will also maximize the produced bnAb titers.

It is of high importance to understand how differences in Ag sequences lead to differences in the types of evolved BCR mutations. An advantage of our model is the ability to gain such molecular level insight by studying in detail the BCR/Ag complexes produced at various stages of AM. This would enable true Ag sequence design, future experimental testing of which would provide the ultimate assessment of how realistic our free energy estimator is for guiding the choice of actual Ags. While beyond the scope of the current work, such analyses and design studies are planned for the future. Continued growth in computing power may also enable future studies with larger B cell population sizes, which may more accurately capture effects of B cell competition and clonal dynamics.

## Methods

We first describe the multiscale model used to simulate the affinity maturation (AM) of a B cell population in a germinal center (GC) in response to vaccination. Following this, details of the analysis protocols are presented.

### Seeding of the germinal center

We assume that a germline (GL)-targeting scheme has occurred prior to vaccination to activate the desired bnAb precursor B cells, in the present case those of the VRC01 class [21]. Experimentally, a few to a hundred B cells have been shown to seed a GC [43]. Due to the sparsity of data currently available of VRC01 class precursor B cells bound to HIV-based antigens (Ags), we modeled the GCs initiated upon the first vaccine immunization as being seeded with a single VRC01 GL precursor B cell [21]. The nucleotide sequence of the B cell receptor (BCR) is input directly into the model, along with the amino acid sequence of the Ag(s) used for vaccination.

### Antigen sequences

Four different Ag sequences were employed in the *in silico* vaccination schemes. These include three Ags (identified as KR, HQ and EU), and the sequence of the wildtype HIV BG505 SOSIP, which is referred to as WT. The three Ags KR, HQ and EU were previously designed by us to have a high potential of eliciting bnAbs to the CD4bs when used in an HIV vaccine [20]. These are based on the WT sequence, with selected mutations introduced from the naturally-occurring Ags KR423280, HQ217523, and EU577271; see original reference for details on the design [20].

### B cell expansion and mutations in the dark zone

After the GC has been seeded, the B cell population undergoes pure expansion (i.e., no mutation/selection) in what is known as the “dark zone” for approximately one week, to reach a population size of 128 cells in the simulation (2^7^). The gene encoding the enzyme activation-induced cytidine deaminase (AID) is then activated, facilitating the accumulation of mutations in the BCRs at a rate of roughly 0.14 mutations per sequence per division [44], with each B cell dividing on average twice per GC cycle [45]. To reduce the cost of the computational modeling, only mutations in the complementarity-determining regions (CDRs) were considered here to influence the BCR/Ag binding free energy (as opposed to considering framework (FRW) mutations as well). As in our previous work [24], we set the probability of a mutation occurring in the CDRs to 0.85. It was also necessary to exclude FRW mutations because our scoring function currently does not account for the possible destabilizing effects of FRW mutations [24]. We also assume that 30% of all mutations are lethal to more closely mimic what has been shown to be the case in experiments [45].

### Mutation model

Mutations in the BCR sequences are introduced by AID on the nucleotide level. Where and which mutations arise in the BCRs during each cycle of the GC reaction is largely a function of the dynamics of AID, which shows pronounced biases towards certain patterns or groups of nucleotides [19]. The tendency of a particular nucleotide in a BCR sequence to be mutated during AM depends primarily on the surrounding microsequence environment of that nucleotide; specifically, it has been shown to depend on the identities of the two neighboring nucleotides on either side in the sequence. [19] Yaari *et al*. considered all possible combinations of such groups of five nucleotides, which they deemed “5-mers”, and determined the relative tendency (“mutability score”) of each 5-mer to be targeted for mutation by comparing the sequences of over 1 million Abs before and after AM [19]. In our model, for a given BCR sequence in a given GC cycle, we first determine all of the relevant 5-mers in a sliding window and look up their corresponding mutability scores. Next, we normalize this subset of mutability scores to convert them into probabilities. They are then used to determine the single nucleotide that will be mutated in the BCR sequence during that GC cycle. In a similar manner as described above, Yaari *et al*. also determined the probability that the central nucleotide in each 5-mer is mutated to each of the other nucleotides (i.e., to A, C, G, or T) [19]. After determining the single nucleotide to undergo mutation in a given BCR/GC cycle, these substitution probabilities are then used in our model to sample the nucleotide.

### Selection, recycling, and differentiation

After acquiring mutations in their receptors, B cells compete with other B cells for binding to the Ags and receiving support from T helper cells [22]. B cells whose receptors are able to bind more strongly to the Ags are in turn able to consume, break down, and display more Ag on their surface, which ultimately increases their chance of receiving productive proliferation signals from T helper cells [22]. B cells that internalize low levels of Ags do not interact productively with T helper cells and undergo apoptosis [46–48]. As in our past work [8], this behavior is modeled by Eq 1:

$$P_{internalize}=\frac{c_{i}∙e^{e_{scale}(E_{i}^{j}-E_{act})}}{1+c_{i}∙e^{e_{scale}(E_{i}^{j}-E_{act})}}$$ (1)

where *c*_*i*_ is the concentration of Ag *i*, $E_{i}^{j}$ is the binding free energy between Ag *i* and B cell *j*, *E*_*act*_ is the activation energy, and *e*_*scale*_ is a pseudo inverse temperature. The activation energy for seeding a GC was chosen to be equal to the weakest binding free energy between the four Ags and VRC01GL–that of EU at -9.01 kcal/mol–such that all four Ags could induce an effective immune response. The value of *e*_*scale*_ (5.8 (kcal/mol)^-1^), along with the value of the breadth threshold (-9.9 kcal/mol; see below), were adjusted such that a single immunization with any of the Ags would produce mostly low-breadth Abs (a breadth ≤ ~0.15). The above condition mimics the fact that breadth is unlikely to result from a single immunization. This is demonstrated by the finding that in natural infection, bnAbs typically require several years to evolve, and that in vaccination studies with mice, they emerge only after several sequential immunizations with variant Ags [7,35]. How the Ag concentrations, *c*_*i*_, used in the simulated immunization schemes were chosen and how the BCR/Ag binding free energies, $E_{i}^{j}$, were calculated are described in the following sections.

B cells that survive this first selection test continue on to compete for T cell help; the top 70% of binders are selected for T cell help. From this pool of B cells that are positively selected, 70% are then chosen randomly to be recycled back to the dark zone for additional rounds of mutation and selection to iteratively increase their affinity for the Ag [23]. The other 30% of B cells exit the GC to simulate differentiation into plasma and memory cells [23].

### Antigen concentration profiles

The Ag concentration used in each successive immunization (c_1_ = 0.25, c_2_ = 0.05, c_3_ = 0.01) was chosen through trial-and-error to maximize the number of protocols for which we could obtain successful results (i.e., GC reactions that terminated due to recovery of the B cell population to its initial size of 128 B cells). In total, 48 protocols were initially considered: 4x3x2 unique 3-Ag protocols, 4x3 unique 2-Ag protocols, and 12 same-Ag protocols (4 3-Ag, 4 2-Ag protocols, and 4 single-Ag protocols). At the final chosen concentrations, we were able to obtain results for 36 of the 48 protocols; for the other 12 protocols, GC reactions did not terminate successfully. For example, after first immunizing with either WT or KR, B cell populations decreased rapidly and did not recover after a second immunization with EU. Thus, we could not obtain results for the following 6 protocols: WT-EU, WT-EU-KR, WT-EU-HQ, KR-EU, KR-EU-WT, or KR-EU-HQ. Also, after first immunizing with HQ, the resultant B cell populations had matured sufficiently such that the B cell populations did not undergo any AM after subsequent administration of WT or HQ. Thus, we also could not obtain results for the following 5 protocols: HQ-WT, HQ-WT-KR, HQ-WT-EU, HQ-HQ, or HQ-HQ-HQ, and for WT-KR-HQ for similar reasons.

### Binding free energy calculations

BCR/Ag binding free energies are computed on-the-fly during the AM simulations, enabled by an efficient pipeline of steps that make use of several pre-existing external resources. First, we convert the mutated nucleotide BCR sequences into amino acid sequences. We then use Modeller [25] to graft these mutated sequences and that of the particular Ag used for immunization onto the crystal structure of VRC01 in complex with an HIV-based Ag [30]. Twelve complexes are independently built with Modeller for each calculation to obtain reliable average estimates for the BCR/Ag binding free energy. Following this conversion of sequence to structure, the Rykunov-Fiser statistical pair potential [26] is used to compute the binding free energy (see S1 Text for more details).

This protocol achieves a good combination of computational efficiency and accuracy: it allows for the computation of one binding free energy in about one minute using a 16-core processor, and shows a Pearson correlation coefficient with experimental binding affinity data of 0.74 (p-value of 1e-9) and an RMSE of 0.59 kcal/mol. The protocol produces results that are comparable to those obtained using all-atom long MD simulations that required hours of time on computing clusters [49], but in a small fraction of the time. We note that the main reason for the high accuracy in our model is fitting to experimental data obtained for similar antibody/antigen complexes (see SI). The number of complexes to model was set to 12 to reduce statistical error in the binding affinity (see Fig G in S1 Text). The number can be reduced or increased further, depending on the computational resources available. In all, for the set of simulations performed in this study, the above protocol was used to calculate the binding free energy of nearly 1 million unique BCR/Ag complexes.

We note that a limitation of the present homology-model-based approach to computing binding affinity changes is that the use of a small number of template structures prevents significant reorientation of the bound antibody. Thus, the present model may not be valid for modeling the maturation of antibodies such as CH103, which has been shown to undergo reorientation upon maturation [28]. However, in the present case of maturation of antibody VRC01, close superposition of the crystallographic coordinates of the germline and mature forms (see Fig H in S1 Text) indicates the same binding pose, which suggests that the present modeling strategy is justified.

### Frustration calculations

The calculated binding free energies were used directly in calculations of the level of frustration imposed on the GC reactions due to vaccination; since we did not change the concentration profiles across the protocols, changes in frustration could be linked directly to changes in Ag sequence or temporal pattern of administration. The frustration of a given immunization was defined as the opposite sign of the average binding free energy of the GC-seeding BCR(s) (germline BCR for 1-Ag protocols; memory BCRs for multi-Ag protocols) against the next-administered Ag, since stronger BCR/Ag binding free energies imposes less frustration by increasing the probability of internalizing Ag (Eq 1). The change in frustration, Δ*F* was then computed between the current and former immunizations (Δ*F*_*t* = 3−*t* = 2_, Δ*F*_*t* = 2−*t* = 1_, and Δ*F*_*t* = 1−*t* = 0_). With regard to the 1-Ag protocols, ‘former’ refers to a reference immunization with frustration set equal to the binding free energy between VRC01GL and the WT Ag at -9.32 kcal/mol, multiplied by -1.

### GC reaction termination

The GC reaction terminates if either: (a) the B cell population recovers to its initial size of 128 B cells in the first immunization or 384 B cells in subsequent immunizations (see next section)–termed a successful reaction–or (b) if all of the B cells die. Simulations were rerun until two successful trials of each protocol were achieved.

### GC reseeding with memory B cells

When multiple immunizations are simulated, the GC for the 2^nd^ and 3^rd^ rounds of affinity maturation are seeded with memory B cells output from the previous immunization. From experimental evidence it is known that the new GC is seeded by a variable number of B cells (tens to hundreds) [43]. In our simulations we used three, chosen at random from the pool of memory B cells. These are then allowed to proliferate in the dark zone without mutation until the B cell population reaches an initial size of 384 B cells (128 x 3). We note that the number of founding B cell clones is higher here than the number of B cells that were chosen to seed a GC upon the first immunization. Since we are not limited by the available experimental data, we increased this number to more accurately model the effects of clonal competition. Increasing this number much beyond three would be computationally intractable with the current model.

### Computing the breadth from the binding free energy

The breadth of a BCR is defined as the fraction of Ags in a panel that can be neutralized by the Ab. We compute the binding breadth *B*, which is based on the binding free energy ΔG of the BCR for all Ags in the panel:

$$B=\frac{1}{N}\sum_{i=1}^{N}\left(\Delta G_{i}<\Delta G_{cutoff}\right)$$ (2)

In this expression, *N* is the number of Ags in the panel, *ΔG*_*i*_ is the binding free energy for the *i*-th Ag, and *ΔG*_*cutoff*_ is an upper limit in the binding free energy above which the BCR is considered to be unable to bind to the Ag [50]. For the panel of Ags, we used the Seaman virus panel from the literature which is composed of 106 Ags [31].

**Fig 5:**
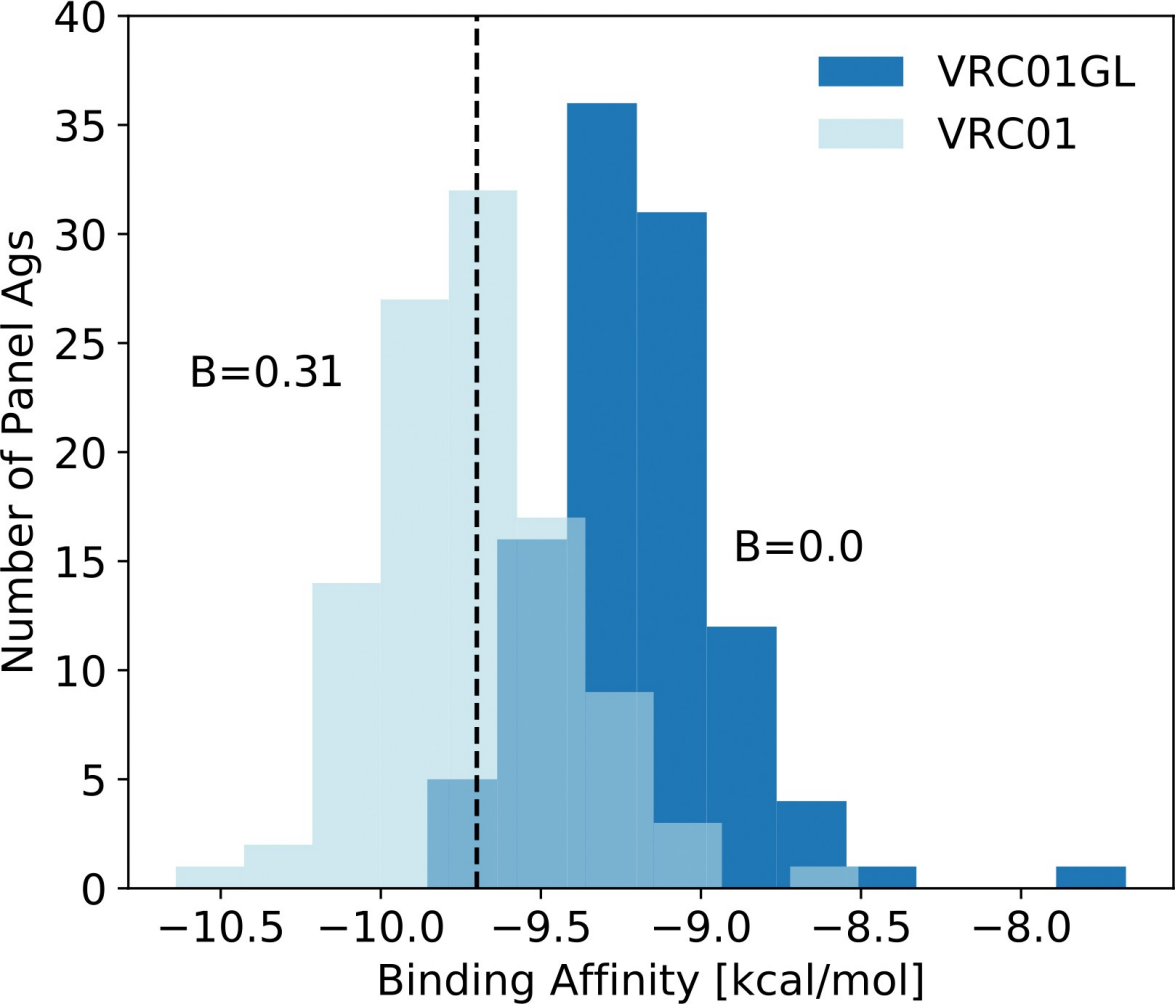
Binding free energy distributions of VRC01GL and VRC01 against a panel of 106 HIV antigens (Ags), using the scoring function developed in this work. A black dashed line indicates the threshold of -9.9 kcal/mol used to determine the breadth (B) of the BCRs. Note that the intermediate-colored region arises from the overlap of the two distributions.

To validate the binding free energy scoring function (see S1 Text for additional validation details), we tested its ability to discriminate between the breadth of the mature VRC01 Ab from its putative germline VRC01GL Ab. This is of the utmost importance as the AM simulations are initiated from the germline BCR sequence, which then evolves over time into a more mature BCR sequence. We computed the binding free energy for VRC01 and VRC01GL for all 106 Ags in the Seaman panel and compared the two distributions (Fig 5). For the calculations, PDB 5FYJ [30] was used as the template. From Fig 5, we can see that the average binding free energy of the germline is shifted to higher values, indicating weaker binding, with a clear separation between the histograms for the mature and germline Abs. Using a threshold binding free energy (B_*TH*_) of -9.9 kcal/mol, the computed breadth of the germline and mature Abs are 0.0 and 0.31, respectively. As shown in Fig 2A and Fig E in S1 Text (top right plot), a threshold of -9.9 kcal/mol also results in a large spread in the mean breadth of the BCRs produced across the different vaccination protocols, allowing us to discriminate between the protocols for features that promote or hinder bnAb formation. If we adopt a less stringent threshold, the difference in breadth is even greater between VRC01GL and VRC01 (e.g., a threshold of -9.7 kcal/mol leads to breadths of 0.0 and 0.64, respectively). However, we observe that the spread in mean BCR breadth across the different vaccination protocols is reduced (Fig E in S1 Text, bottom plots), with many protocols nearing the saturation limit (mean breadth of 1.0). Likewise, while the spread in mean BCR breadth is slightly increased using a more stringent threshold than -9.9 kcal/mol (Fig E in S1 Text, top left plot), this leads to poorer discrimination in breadth between VRC01GL and VRC01. Thus, -9.9 kcal/mol represents an optimal choice for the breadth threshold.

### *In silico* BCR (CD4bs) interfacial residue definition

**Fig 6:**
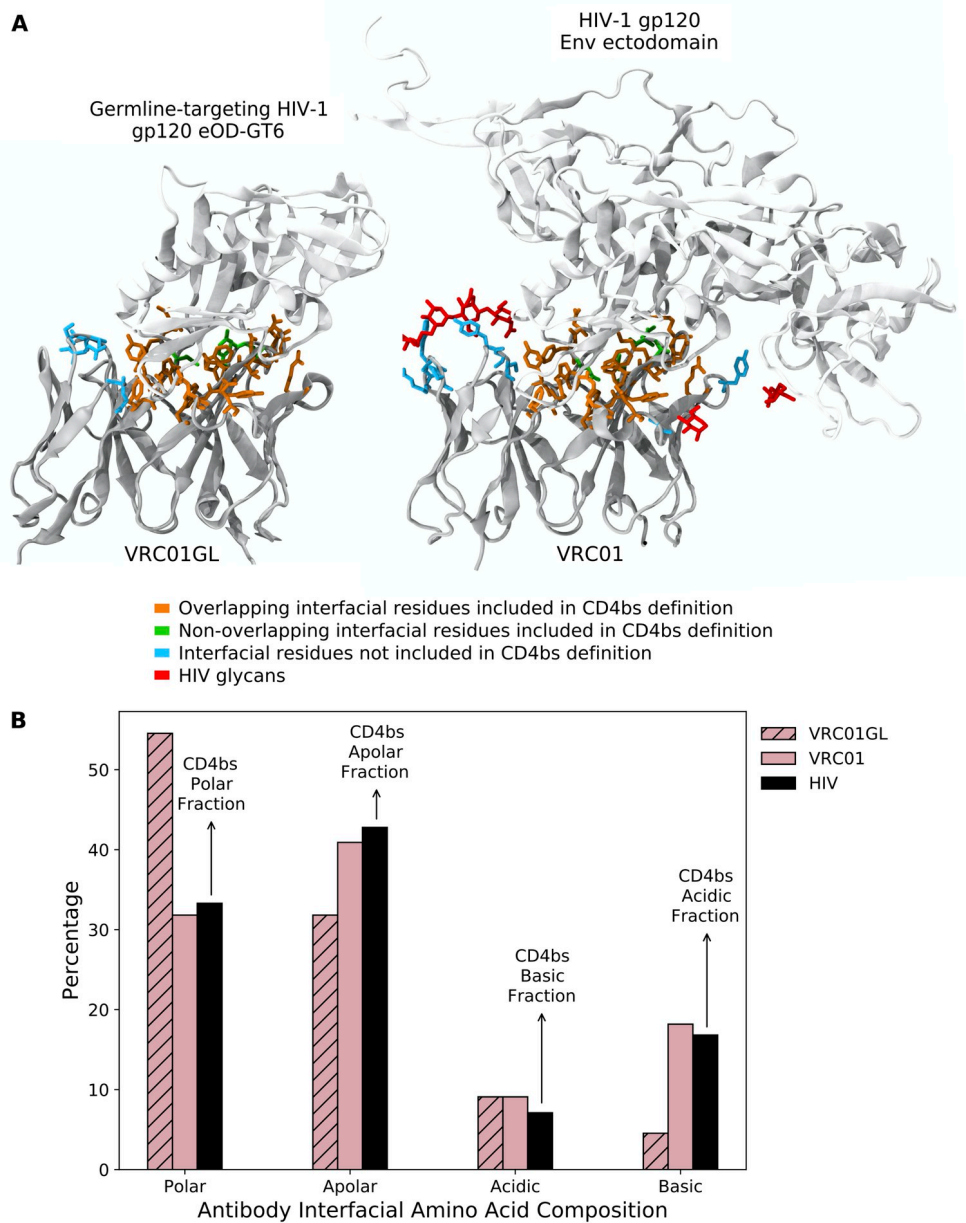
Definition of interfacial BCR residues used to characterize AM simulations. (A) VMD snapshots of (left) VRC01GL and (right) VRC01, in complex with HIV-based Ags. Ags are colored in silver and Abs in gray. Residues included in the *in silico* CD4bs interfacial residue definition are colored orange if overlapping between the two Abs and colored green otherwise, and interfacial residues excluded from the definition due to contact or alignment with HIV glycans (red) are colored cyan. (B) Interfacial amino acid compositions for VRC01GL and VRC01 are shown by hatched and solid pink bars, respectively, using the residues defined in orange/green in (A), which mimic the biological trends shown in Fig 3. For each amino acid type, the Abs’ target interfacial fraction in the CD4bs of HIV is indicated by a black bar (note that the Abs’ acidic interfacial compositions evolve to match the basic interfacial composition of the CD4bs, and vice versa).

In the crystal structure of VRC01 in complex with HIV gp120, nine residues were found to be in direct contact (within 4Å) with glycans on the surface of the Ag (Fig 6A, right). Our scoring function for the BCR/Ag binding free energy was not parameterized to include the effect of glycans. Thus, we removed the glycan moieties from the structure files prior to the calculations and excluded these residues from our CD4bs interfacial Ab residue definition, which was used to determine the degree of interfacial composition matching of the BCRs from the AM simulations. We note that inclusion of the 11 glycan-contacting residues in our calculations would lead to incorrect assessments of the degree of ICM achieved by the produced BCRs, because the driving force to evolve certain mutations against HIV glycans is not captured by our model. We also excluded an additional two interfacial residues in VRC01GL that positionally aligned with these nine residues (Fig 6A, left). All other interfacial residues identified for VRC01GL and VRC01 were considered for a total of 22 residues (see Table B in S1 Text). Despite the above residue exclusions, as shown in Fig 6B, the set of 22 interfacial residues is sufficient to capture the major trends in composition matching observed in Fig 3, namely large increases in the basic and apolar interfacial fractions and a large decrease in the polar interfacial fraction. Amino acids were classified as follows: polar (S, T, C, Y, N, Q), apolar (G, A, V, L, I, M, W, F, P), acidic (D, E), and basic (K, R, H).

BCR interfacial residues in contact with conserved Ag residues were similarly identified using Visual Molecular Dynamics (VMD [51]). A total of 15 Ab residues were identified to be within 4Å of one of eight conserved Ag residues in PDB code 5FYJ (see Table C in S1 Text). These 15 residues represent 68% of the 22 total BCR interfacial residues listed in Table B in S1 Text. The conserved Ag residues were identified by Conti et al. [20] as those for which the cost of mutational escape is high, according to the fitness landscape of HIV gp160 [51]. Consequently, these residues are generally highly conserved across many HIV strains and are commonly targeted by bnAbs against the CD4bs [20]. For each immunization, we counted the number of BCR mutations that had arisen at one of the 15 identified residue positions for each produced B cell clone to determine the extent of mutation towards conserved Ag sites. The results were then weighted by the size of each B cell clone and averaged across the two independent simulation trials (except for the KR-KR-KR and HQ-EU-WT protocols, for which only one successful trial could be obtained).

### Interfacial composition/electrostatic pattern matching (ICM) calculations

For the ICM analysis of anti-CD4bs bnAbs (see Results), the following crystallographic structures were used: PDB codes 4JPK and 5FYJ for the germline [21] and mature [30] forms of VRC01, respectively, and PDB codes 5IGX and 3U7Y for the germline [32] and mature [33] forms of NIH45-46, respectively. Only the structure of the germline [32] Ab is currently available for 3BNC60 (PDB code 5FEC), whereas only the structure of the mature [34] Ab is available for CH103 (PDB code 4JAN). To overcome the challenge of missing structural data, we noted that the binding interface for VRC01 and NIH45-46 changes very little upon Ab maturation. Assuming that this holds for the other Abs, the same structure was used for the germline and mature forms of 3BNC60 and CH103 to identify interfacial residues in each case.

For the ICM analysis of the HIV CD4bs composition, interfacial Ag residues within 4Å of any Ab residue were identified from the crystal structures of multiple bnAbs in complex with HIV gp120, namely two structures of VRC01 (PDB codes 5FYJ [30] and 3NGB [52]), and one structure each of VRC18 (PDB code 4YDL [53]) and NIH45-46 (PDB code 3U7Y [33]). Sixteen common Ag interfacial residue indices were then identified across all of these data sets to isolate only the most conserved sites involved in Ab binding. These sites were used to determine the ICM of each of the 106 Ags on the Seaman virus panel [31], and the results were averaged across the panel.

The equation for calculating the ICM score for the BCRs in a single B cell clone *i* is shown below:

$${ICM}_{i}=1-(\frac{{\left[\chi_{i,P}-\chi_{Ag,P}\right]}^{2}}{\chi_{Ag,P}}+\frac{{\left[\chi_{i,Ap}-\chi_{Ag,Ap}\right]}^{2}}{\chi_{Ag,Ap}}+\frac{{\left[\chi_{i,Ac}-\chi_{Ag,B}\right]}^{2}}{\chi_{Ag,B}}+\frac{{\left[\chi_{i,B}-\chi_{Ag,Ac}\right]}^{2}}{\chi_{Ag,Ac}})$$ (3)

where *P*, *Ap*, *Ac*, and *B* stand for polar, apolar, acidic, and basic amino acid types, respectively, *χ*_*i*,*k*_ is the interfacial fraction of amino acid type *k* for clone *i*, and *χ*_*Ag*,*k*_ is the average interfacial fraction of amino acid type *k* for the Ag panel. As such, ICM is simply a quantity that reaches a maximum of 1.0 for a perfect match against a target interfacial amino acid composition, with smaller values indicating worse agreement.

The ICM for an AM simulation is then defined as the average of the ICM scores of all individual clones (*ICM*_*i*_), weighted by the number of B cells in each clone (*N*_*i*_):

$$ICM=\frac{1}{\sum_{i}N_{i}}\sum_{i}{N_{i}\;ICM}_{i}$$ (4)

## Supporting information

S1 Text Figure and table captions. *Table A in S1 Text*: Simulated vaccination protocols with 1, 2, or 3 sequentially administered Ags. Results are shown for the mean BCR breadth, mean degree of interfacial composition/electrostatic pattern matching (ICM), and the mean number of GC cycles. Error bars represent the standard deviation of two independent simulations of each vaccination protocol (except for the KR-KR-KR and HQ-EU-WT protocols, for which only one successful trial could be obtained), consisting of between one and three immunizations/GC reactions. Note that no error bars exist for mean frustration since a single calculation of binding free energy was carried out for any given BCR/Ag complex. *Table B in S1 Text*: List of 22 amino acid residues used in the *in silico* CD4bs interfacial BCR residue definition. Residue numbers and identities correspond to those in PDB codes 5FYJ [30] for VRC01 and 4JPK [21] for VRC01GL. *Table C in S1 Text*: List of conserved residues in the CD4bs of HIV, as determined by Conti et al. [20], and the corresponding residues of VRC01 in contact with the Ag residues, used to characterize BCR conserved site binding (Fig 2D, main text). Residue numbers and identities correspond to those in PDB code 5FYJ [30]. *Fig A in S1 Text*: Snapshots from Visual Molecular Dynamics (VMD [54]) of VRC01 (left) and CH103 (right) in complex with gp120-based Ags. Ags are shown in pink and Abs in transparent gray, with the six complementarity-determining regions (CDRs) colored in red (CDRH2), orange (CDRL2), yellow (CDRH3), green (CDRL3), blue (CDRH1), and purple (CDRL1). *Fig B in S1 Text*: Basic biological principles govern the mutations acquired by VRC01 during AM. (A) Heat map of the fraction of interfacial mutations (n = 14) made from each amino acid (AA) type in VRC01GL to each AA type in VRC01. The overall mutation tendency for each AA type, *μ*, is computed as the sum of the relevant column values subtracted by the sum of the relevant row values. (B) Heat map of the average number of nucleotides needed to transition from one AA type to another. (C) Mean mutability of the different AA types, based on the model of Yaari *et al*. [19]. (D) Mutability score of each nucleotide in VRC01GL, grouped and colored by the type of AA encoded by the codon to which the nucleotide belongs. *Fig C in S1 Text*: Mean BCR breadth of vaccination protocols with t = 1, 2, and 3 single-Ag sequential immunizations versus the mean weighted degree of interfacial composition and electrostatic pattern matching (ICM), computed using different thresholds for determining the BCR breadth. Error bars are only shown for the degree of ICM for clarity and represent the standard deviation of two independent simulations of each vaccination protocol (except for the KR-KR-KR and HQ-EU-WT protocols, for which only one successful trial could be obtained), consisting of between one and three immunizations/GC reactions. *Fig D in S1 Text*: Fraction of different aa types at the BCR/Ag interface in BCR sequences produced after administration of protocols beginning with (A) the EU Ag, (B) the KR Ag, (C) the WT Ag, or (D) the HQ Ag. Black dashed lines indicate the interfacial amino acid fractions in the CD4bs of HIV. “Immunization 0” refers to the GL-targeting scheme that we assume takes place prior to vaccination (see main text). Error bars represent the standard deviation of two independent simulations of each vaccination protocol (except for the KR-KR-KR and HQ-EU-WT protocols, for which only one successful trial could be obtained), consisting of between one and three immunizations/GC reactions. *Fig E in S1 Text*: Effect of changing the breath threshold (B_*TH*_) on the distribution in breadth of the BCRs produced from AM simulations of various vaccination protocols. Mean BCR breadth of vaccination protocols with t = 1, 2, and 3 single-Ag sequential immunizations for a breadth threshold of -10.0 kcal/mol (top left), -9.9 kcal/mol (top right; value used in AM simulations), -9.8 kcal/mol (bottom left), and -9.7 kcal/mol (bottom right). Error bars represent the standard deviation of two independent simulations of each vaccination protocol (except for the KR-KR-KR and HQ-EU-WT protocols, for which only one successful trial could be obtained), consisting of between one and three immunizations/GC reactions. *Fig F in S1 Text*: Linear correlation between experimental and computed binding affinities using the RF_HA_SRS statistical pair potential. Each dot represents one binding affinity value. The data in orange are used for the training of the linear regression, while in blue for the validation. *Fig G in S1 Text*: Standard error in the computed binding affinity values as a function of the number of structural models for the VRC01 and VRC01GL antibodies bound to the wild-type (WT) antigen. *Fig H in S1 Text*: Comparison between the binding pose of VRC01 and its germline VRC01GL from experimental structures (PDB: 3NGB and 4JPK, respectively). In blue and cyan is the antigen (AG), in red and pink are the antibody heavy chains (HC), and in lime and green are the antibody light chains (LC), for VRC01 and VRC01GL, respectively. *Fig I in S1 Text*: A) Relative binding affinity changes while mutating the 25 residues of VRC01GL at the binding site into each possible amino acid, sorted by magnitude. The range of the magnitudes is as expected limited with most mutations neutral (close to zero). B) Bar plot of the probability that mutating to a given amino acid improves the binding affinity. There are no preferred amino acids with very high percentages.
(PDF)
