# Supplementary material for: Multiscale affinity maturation simulations to elicit broadly neutralizing antibodies against HIV
Source: PLoS Comput Biol. 2022 Apr 20;18(4):e1009391. doi: 10.1371/journal.pcbi.1009391 (PMC9020693; doi:10.1371/journal.pcbi.1009391)
Supplement: S1 Text — Table A in S1 Text: Simulated vaccination protocols with 1, 2, or 3 sequentially administered Ags. Results are shown for the mean BCR breadth, mean degree of interfacial composition/electrostatic pattern matching (ICM), and the mean number of GC cycles. Error bars represent the standard deviation of two independent simulations of each vaccination protocol (except for the KR-KR-KR and HQ-EU-WT protocols, for which only one successful trial could be obtained), consisting of between one and three immunizations/GC reactions. Note that no error bars exist for mean frustration since a single calculation of binding free energy was carried out for any given BCR/Ag complex. Table B in S1 Text: List of 22 amino acid residues used in the in silico CD4bs interfacial BCR residue definition. Residue numbers and identities correspond to those in PDB codes 5FYJ [30] for VRC01 and 4JPK [21] for VRC01GL. Table C in S1 Text: List of conserved residues in the CD4bs of HIV, as determined by Conti et al. [20], and the corresponding residues of VRC01 in contact with the Ag residues, used to characterize BCR conserved site binding (Fig 2D, main text). Residue numbers and identities correspond to those in PDB code 5FYJ [30]. Fig A in S1 Text: Snapshots from Visual Molecular Dynamics (VMD [54]) of VRC01 (left) and CH103 (right) in complex with gp120-based Ags. Ags are shown in pink and Abs in transparent gray, with the six complementarity-determining regions (CDRs) colored in red (CDRH2), orange (CDRL2), yellow (CDRH3), green (CDRL3), blue (CDRH1), and purple (CDRL1). Fig B in S1 Text: Basic biological principles govern the mutations acquired by VRC01 during AM. (A) Heat map of the fraction of interfacial mutations (n = 14) made from each amino acid (AA) type in VRC01GL to each AA type in VRC01. The overall mutation tendency for each AA type, μ, is computed as the sum of the relevant column values subtracted by the sum of the relevant row values. (B) Heat map of the average number of nucleot [file pcbi.1009391.s001.pdf]

SUPPORTING INFORMATION

Multiscale affinity maturation simulations to elicit broadly neutralizing antibodies against HIV

Simone Conti [1], Victor Ovchinnikov[1], Jonathan G. Faris[2], Arup K. Chakraborty[3,4,5,6,7], Martin Karplus[1,8], and Kayla G. Sprenger[2*]*

[1]Department of Chemistry and Chemical Biology, Harvard, Cambridge, Massachusetts; [2]Department of Chemical and Biological Engineering, University of Colorado Boulder, Boulder, Colorado; [3]Institute for Medical Engineering and Science, Massachusetts Institute of Technology (MIT), Cambridge, Massachusetts; [4]Department of Chemical Engineering, MIT, Cambridge, Massachusetts; [5]Department of Physics, MIT, Cambridge, Massachusetts; [6]Ragon Institute of MGH, MIT and Harvard, Cambridge, Massachusetts; [7]Department of Chemistry, MIT, Cambridge, Massachusetts; [8]Laboratoire de Chimie Biophysique, Institut de Science et d'Ingénierie Supramoléculaires, Université de Strasbourg, Strasbourg, France

* kayla.sprenger@colorado.edu (KGS); * marci@tammy.harvard.edu (MK)

Table A. Simulated vaccination protocols with 1, 2, or 3 sequentially administered Ags. Results are shown for the mean BCR breadth, mean degree of interfacial composition/electrostatic pattern matching (ICM), and the mean number of GC cycles. Error bars represent the standard deviation of two independent simulations of each vaccination protocol (except for the KR-KR-KR and HQ-EU-WT protocols, and the WT-WT-WT null model simulation, for which only one successful trial could be obtained), consisting of between one and three immunizations/GC reactions. Note that no error bars exist for mean frustration since a single calculation of binding free energy was carried out for any given BCR/Ag complex.

#	Protocol	Mean Breadth	Mean ICM	Mean GC Cycles	Mean Frustration
**1**	WT	0.00±0.00	0.75±0.00	7.5±0.5	9.32
	WT (null model)	0.08±0.04	0.80±0.02	12±3.0	9.32
**2**	KR	0.00±0.01	0.79±0.05	12±0.5	9.28
**3**	HQ	0.15±0.03	0.83±0.09	14±0.5	9.15
**4**	EU	0.11±0.00	0.71±0.03	14±1.5	9.01
**5**	WT-WT	0.38±0.06	0.86±0.01	24±3.0	9.30
	WT-WT (null model)	0.36±0.20	0.84±0.07	20±0.5	9.30
**6**	WT-KR	0.65±0.09	0.86±0.06	26±1.0	9.13
**7**	WT-HQ	0.56±0.02	0.83±0.08	24±3.0	9.12
**8**	KR-KR	0.60±0.12	0.92±0.01	38±5.5	9.22
**9**	KR-WT	0.33±0.04	0.84±0.05	32±0.5	9.31
**10**	KR-HQ	0.54±0.08	0.73±0.07	42±2.5	9.14
**11**	HQ-KR	0.33±0.10	0.88±0.03	21±2.0	9.61
**12**	HQ-EU	0.34±0.07	0.88±0.03	21±3.0	9.59
**13**	EU-EU	0.59±0.21	0.82±0.03	25±0.0	9.28
**14**	EU-WT	0.12±0.01	0.71±0.03	18±2.0	9.50
**15**	EU-KR	0.20±0.04	0.72±0.03	22±2.5	9.45
**16**	EU-HQ	0.42±0.14	0.76±0.04	24±0.5	9.41
**17**	WT-WT-WT	0.49±0.002	0.86±0.01	30±5.5	9.75
	WT-WT-WT (null model)	0.59±N/A	0.92±N/A	25±N/A	9.75
**18**	WT-KR-EU	0.83±0.03	0.86±0.08	32±4.5	9.88
**19**	WT-HQ-EU	0.92±0.05	0.84±0.07	34±2.0	9.76
**20**	WT-HQ-KR	0.64±0.04	0.84±0.08	29±1.0	9.79
**21**	KR-KR-KR	0.92±N/A	0.79±N/A	68±N/A	9.50
**22**	KR-WT-HQ	0.65±0.15	0.89±0.02	42±1.5	9.56
**23**	KR-WT-EU	0.76±0.15	0.83±0.03	54±2.5	9.52
**24**	KR-HQ-WT	0.29±0.18	0.75±0.10	46±2.5	9.83
**25**	KR-HQ-EU	0.89±0.05	0.84±0.08	55±1.0	9.61
**26**	HQ-KR-WT	0.44±0.19	0.88±0.03	30±5.5	9.80
**27**	HQ-KR-EU	0.80±0.16	0.89±0.03	49±11.0	9.57
**28**	HQ-EU-WT	0.83±N/A	0.85±N/A	36±N/A	9.84
**29**	HQ-EU-KR	0.84±0.02	0.88±0.05	36±6.5	9.73
**30**	EU-EU-EU	0.90±0.05	0.83±0.03	51±3.0	9.54
**31**	EU-WT-KR	0.94±0.01	0.84±0.07	38±0.5	9.58
**32**	EU-WT-HQ	0.81±0.13	0.80±0.10	42±3.5	9.54
**33**	EU-KR-WT	0.69±0.04	0.81±0.05	40±0.5	9.67
**34**	EU-KR-HQ	0.87±0.06	0.88±0.04	39±3.0	9.58
**35**	EU-HQ-WT	0.49±0.12	0.77±0.08	30±4.5	9.88
**36**	EU-HQ-KR	0.82±0.13	0.81±0.11	36±7.0	9.74

Table B. List of 22 amino acid residues used in the *in silico* CD4bs interfacial BCR residue definition. Residue numbers and identities correspond to those in PDB codes 5FYJ [1] for VRC01 and 4JPK [2] for VRC01GL.

#	Residue	VRC01GL	VRC01	#	Residue	VRC01GL	VRC01
**1**	33	Y	T	**12**	60	A	A
**2**	47	W	W	**13**	61	Q	R
**3**	50	W	W	**14**	64	Q	Q
**4**	52	N	K	**15**	71	R	R
**5**	53	N	R	**16**	99	D	D
**6**	54	S	G	**17**	100	Y	Y
**7**	55	G	G	**18**	100	N	N
**8**	56	G	A	**19**	100	W	W
**9**	57	T	V	**20**	91	Y	Y
**10**	58	N	N	**21**	96	E	E
**11**	59	Y	Y	**22**	97	F	F

Table C. List of conserved residues in the CD4bs of HIV, as determined by Conti et al. [3], and the corresponding residues of VRC01 in contact with the Ag residues, used to characterize BCR conserved site binding (Fig 2D, main text). Residue numbers and identities correspond to those in PDB code 5FYJ [1].

#	Conserved Ag Residue	VRC01 Contacting Residues
**1**	D457	W47, N58, Y59, C60, R61, Q64
**2**	G458	W47, N58, Y59, C60, R61, $E96_L$, $F97_L$
**3**	C459*	W47, C60, R61, $E96_L$, $F97_L$
**4**	S365	A56, V57, N58, Y59, Q64
**5**	G366	G54, G55, A56, V57, Y59
**6**	G367	G54, G55, A56, V57, R71
**7**	D368	R53, G54, G55, A56, R71
**8**	G473	R53, G54, A56, K52

*Note that in BG505, this residue is a glycine (G)
A subscript of 'L' indicates light chain residues

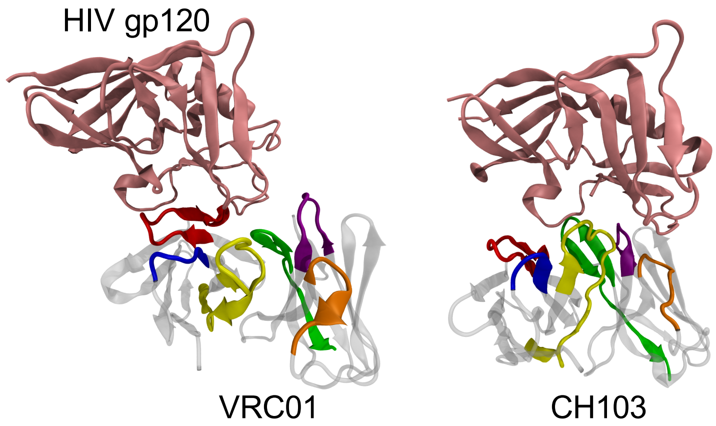

Fig A. Snapshots from Visual Molecular Dynamics (VMD [4]) of VRC01 (left) and CH103 (right) in complex with gp120-based Ags. Ags are shown in pink and Abs in transparent gray, with the six complementarity-determining regions (CDRs) colored in red (CDRH2), orange (CDRL2), yellow (CDRH3), green (CDRL3), blue (CDRH1), and purple (CDRL1).

Four key biological principles dictate the types of observed mutations

Our results establish interfacial composition/electrostatic pattern matching as a major biological driving force in the affinity maturation trends of anti-CD4bs bnAbs. To gain insight into other factors that might influence the evolutionary trajectories of these Abs, we examined in more detail the specific interfacial mutations that VRC01GL made upon maturing into its bnAb form. We considered 33 interfacial residue indices in this analysis, including the 21 overlapping residue indices between VRC01GL and VRC01, as well as the 4 additional residue indices identified solely in VRC01GL and 8 additional residue indices identified solely in VRC01. Of these 33 residues, 14 were mutated during the evolution of VRC01GL into VRC01. We characterized each of these 14 mutations according to which amino acid (AA) type it was mutated from/to in VRC01GL and VRC01, respectively, and then determined the overall fraction of mutations from/to each AA type. These results are presented in Fig B, subplot A, as a heat map.

In line with the results of Fig 3 (main text), the results of Fig B, subplot A, show that a substantial fraction of the interfacial mutations was made away from polar AAs. This is true even considering the large fraction of mutations made away from polar AAs simply to other

polar AAs. We sum up the fraction of mutations made away from polar AAs (Fig B, subplot A, top row) and subtract this from the summed fraction of mutations made to polar AAs (Fig B, subplot A, far left column), to see that the overall selection tendency for polar AAs at the interface, or μ_{Polar}, is strongly negative (-0.50). Similarly, we find a strong positive selection tendency towards apolar (μ_{Apolar} =+0.15) and basic (μ_{Basic} =+0.28) AAs at the interface, also in line with the results of Fig 3 (main text). However, the same analysis shows a mild positive selection tendency towards acidic residues (μ_{Acidic} =+0.07), seemingly at odds with the results of Fig 3. In VRC01GL, 3/25 interfacial residues are acidic, compared to 3/29 residues in VRC01, resulting in the decreased interfacial acidic fraction in Fig 3. For one of the acidic interfacial residues in VRC01, its identity in VRC01GL was an apolar residue, explaining the observed tendency to mutate towards acidic residues in Fig B, subplot A.

These results raise a series of questions, such as why the need for increasing the basic interfacial fraction was met primarily through making mutations away from polar residues versus away from apolar residues, or why so many seemingly unessential polar-to-polar and apolar-to-apolar transitions occurred. Answers to these questions are in part obtained by considering the average number of nucleotides required to transition from one type of AA to another. These values are shown in Fig B, subplot B, and were obtained by calculating the average pairwise distance between all codons encoding for each pair of AA types. From Fig B, subplot B, we can see that fewer nucleotides are required on average to make a polar-to-basic transition than to make an apolar-to-basic transition. Also, given a certain AA, the data shows that it is easiest to mutate to a different AA in the same category, due to the similarities in their codons. This explains, at least in part, the many polar-to-polar and apolar-to-apolar mutations that were made during the evolution of VRC01. Another contributing factor for these mutations is that many polar-to-polar and apolar-to-apolar mutations are "neutral" mutations, which are neither positively nor negatively selected during AM. It is known that VRC01-class Abs typically emerge only after many years of infection [5,6], and so the Abs have long maturation periods over which to accumulate these largely irrelevant mutations. The reasons for the lack of basic-to-basic and acidic-to-acidic mutations – or any mutations away from charged AAs, for that matter – are two-fold. We can observe from Fig 3 (main text) that charged AA types make up the lowest interfacial fractions by far in VRC01GL and

thus they are much less likely to be targeted for mutation, simply from a probability standpoint.

In addition, nucleotides in codons encoding for polar AAs are also, on average, marginally more mutable than those in codons that encode for the other AA types (Fig B, subplot C). However, it is important to note that the fluctuations are the same order of magnitude as the averages and increase as the averages increase, hence differences in codon mutability are likely only a weak driving force for the types of mutations acquired by VRC01 during AM. The values reported in Fig B, subplot C, were determined using the model developed by Yaari *et al*. [7] (see Methods in main text). We arrived at the values reported in Fig B, subplot C, by computing the average mutability score of all 5-mers containing a given codon for all codons encoding for a particular AA type. Computing the mutability scores for the individual nucleotides in the CDRs of VRC01GL in a similar manner as described above, Fig B, subplot D, shows that there are many more nucleotides in codons that encode for polar AAs – and even apolar AAs – with high mutability scores, than there are basic or acidic AAs with high mutability scores (e.g., 17 data points classified as polar have a score > 2, versus 6, 2, and 1 data points classified as apolar, basic, and acidic, respectively).

To summarize, four key biological principles dictate the types of observed mutations in VRC01 as compared with VRC01GL. These are the mutations that we expect to observe in our simulations. From a targeting standpoint, both the initial, relative proportions of different AA types at the interface (principle #1) and the mutability of the codons encoding for the interfacial AAs (principle #2), have a large impact on the evolved mutations. Moreover, mutational patterns are heavily influenced by the difficulty of transitioning from one AA type to another, which determine the length of time required for the GC reactions to take place (principle #3). Lastly, from a selection standpoint, a large initial difference between the interfacial AA composition of the germline BCR and that of the CD4bs, creates a strong driving force to evolve mutations that lead to increased interfacial composition/electrostatic pattern matching (principle #4). The degree of correlation between these principles and how changes in the parameters impact the ensuing number and type of acquired Ab mutations will be explored in detail in future studies (e.g., the impact on AM of using germline sequences with different initial mutability patterns or degrees of interfacial composition matching). Here, we explore

the impact of administering multiple variant Ags in different temporal patterns on the ability of the BCR population to evolve mutations that (1) lead to high breadth, and (2) lead to an increased degree of interfacial composition/electrostatic pattern matching against the CD4bs.

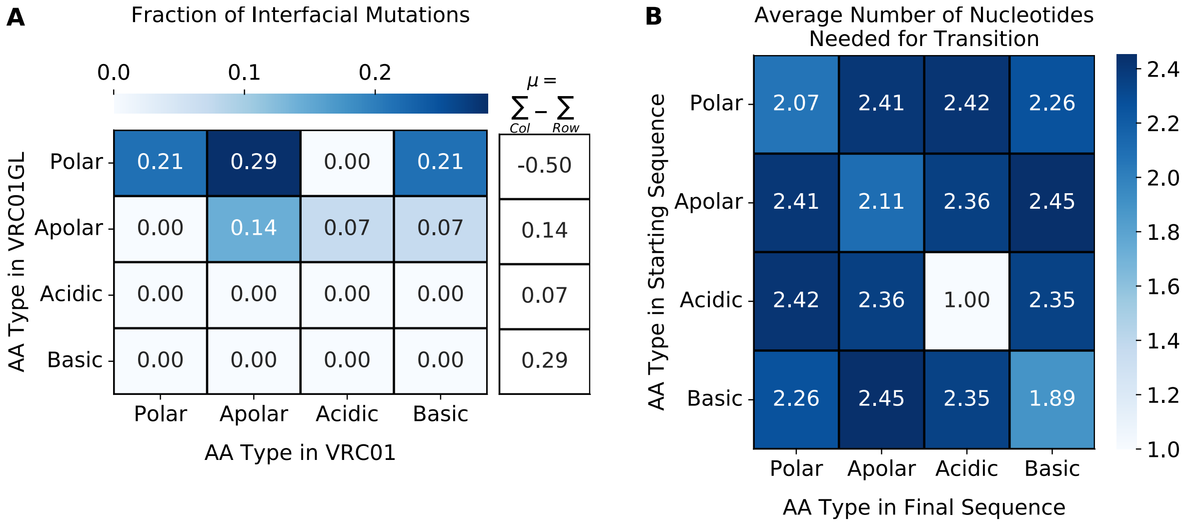

C

AA Type	Mean Mutability Score
Polar	1.10 +/- 0.86
Apolar	0.97 +/- 0.88
Basic	0.92 +/- 0.79
Acidic	0.77 +/- 0.63

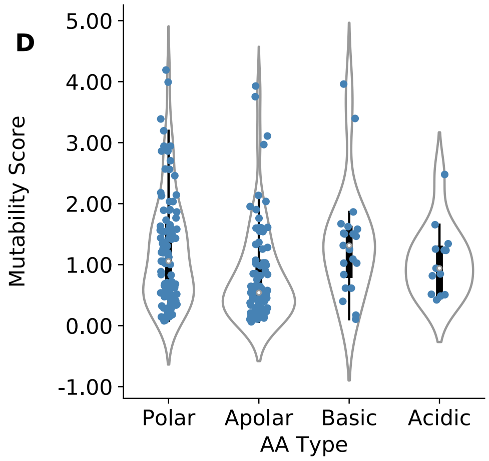

Fig B. Basic biological principles govern the mutations acquired by VRC01 during AM. (A) Heat map of the fraction of interfacial mutations (n=14) made from each amino acid (AA) type in VRC01GL to each AA type in VRC01. The overall mutation tendency for each AA type, μ, is computed as the sum of the relevant column values subtracted by the sum of the relevant row values. (B) Heat map of the average number of nucleotides needed to transition from one AA type to another. (C) Mean mutability of the different AA types, based on the model of Yaari *et al.* [7]. (D) Mutability score of each nucleotide in VRC01GL, grouped and colored by the type of AA encoded by the codon to which the nucleotide belongs.

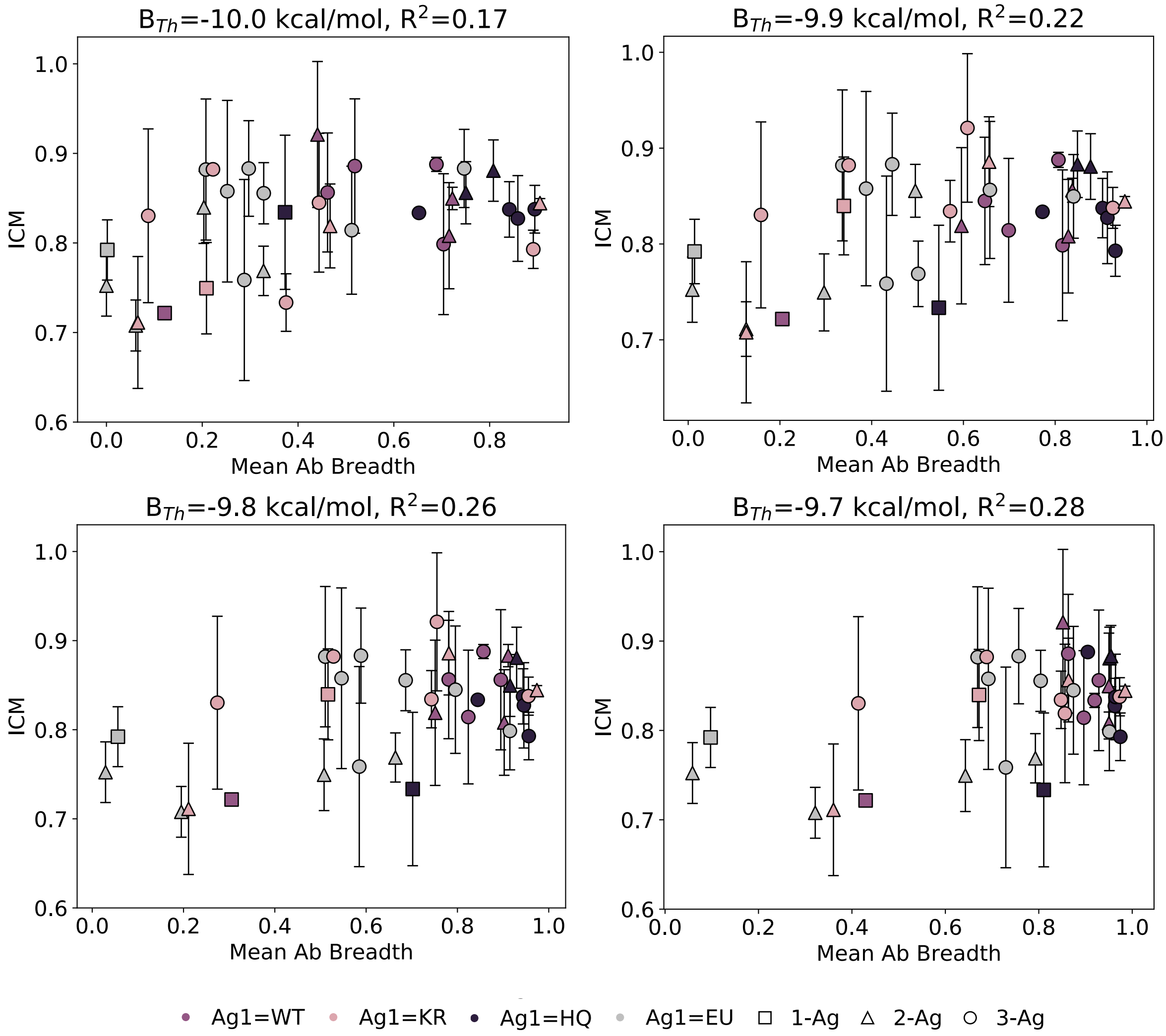

Fig C. Mean BCR breadth of vaccination protocols with t=1, 2, and 3 single-Ag sequential immunizations versus the mean weighted degree of interfacial composition and electrostatic pattern matching (ICM), computed using different thresholds for determining the BCR breadth. Error bars are only shown for the degree of ICM for clarity and represent the standard deviation of two independent simulations of each vaccination protocol (except for the KR-KR-KR and HQ-EU-WT protocols, for which only one successful trial could be obtained), consisting of between one and three immunizations/GC reactions.

Analysis of mutational trajectories of individual clonal BCR sequences

The results for the EU-based protocols (Fig D, subplot A) and HQ-based protocols (Fig D, subplot D) were discussed in the main text. For the KR-based protocols (Fig D, subplot B), we observe that BCR populations collectively mutate to decrease the fraction of polar residues at the interface and increase the fraction of basic residues at the interface after both the first and second immunizations, as was the case with the HQ-based protocols (note, these results do not imply polar-to-basic mutations occurred within the same BCR or even within the same clone in a given round of AM). These results shed light on the high ICM score of the KR-KR protocol, as mentioned earlier in the main text. However, after a third immunization with KR, we observe that the BCR population collectively mutates to revert back to the polar and basic interfacial fractions – and the lower ICM score – obtained after the first immunization with KR. For the rest of the KR-based protocols, no clear trends exist in the types of population-level mutations that are evolved after the third immunization, providing a likely explanation for the wide variation in final ICM scores reported in Fig 4 (main text).

For the WT-based protocols (Fig D, subplot C), the first immunization resulted in little change in the interfacial amino acid fractions of the resulting BCR populations as compared with VRC01GL. After the second immunization, regardless of which Ag was administered, we observe relatively large decreases and increases in the polar and basic interfacial fractions of the produced BCRs on the population level, respectively. However, these ICM gains are offset by mutations that decreased the apolar interfacial fraction for all four WT-based protocols. The third and final immunization, again regardless of which Ag was administered, generally led to little change in the interfacial fraction of any amino acid type, resulting in the intermediate ICM scores for the 2- and 3-Ag WT-based protocols shown in Fig 4 (main text).

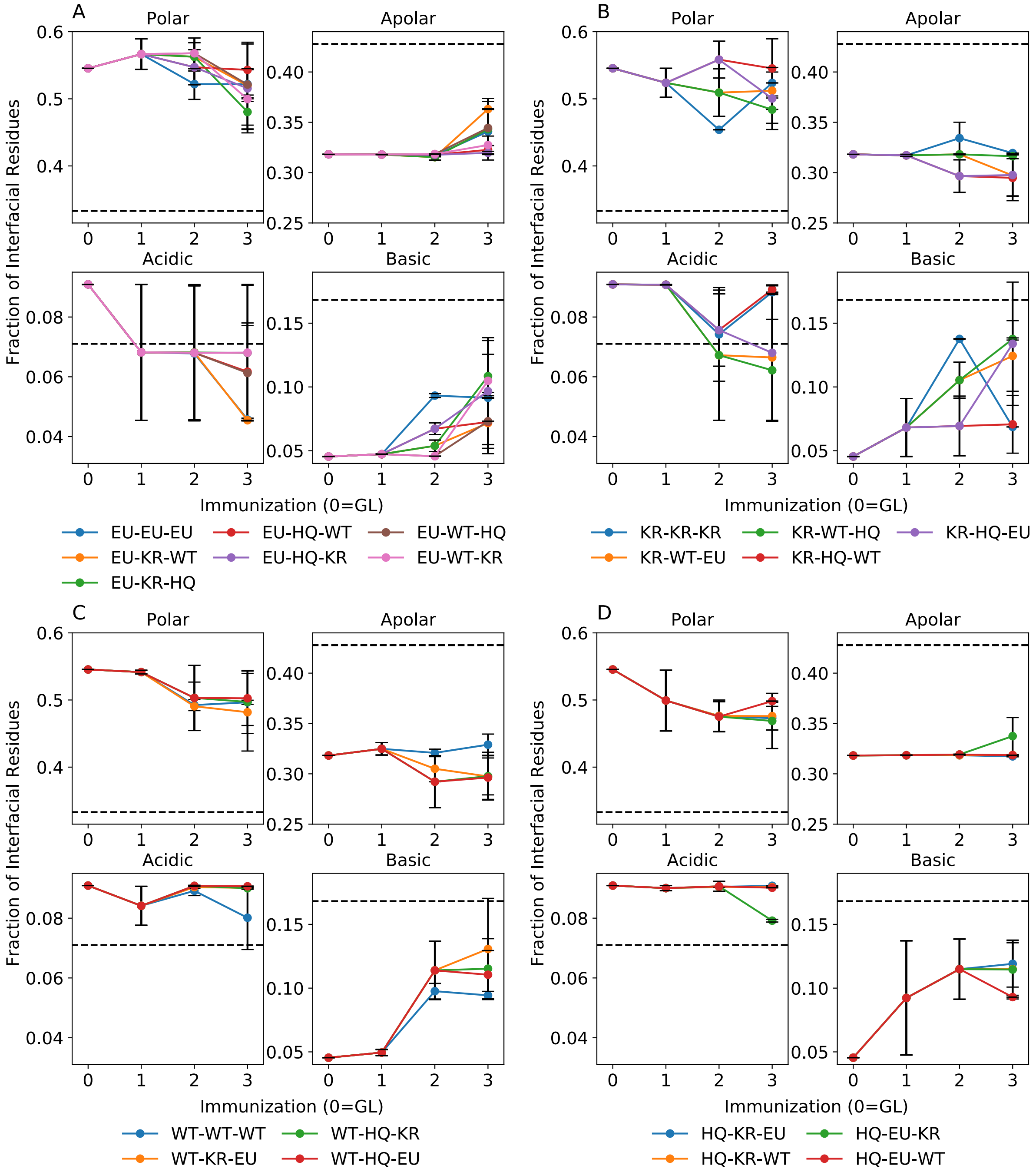

Fig D. Fraction of different aa types at the BCR/Ag interface in BCR sequences produced after administration of protocols beginning with (A) the EU Ag, (B) the KR Ag, (C) the WT Ag, or (D) the HQ Ag. Black dashed lines indicate the interfacial amino acid fractions in the CD4bs of HIV. "Immunization 0" refers to the GL-targeting scheme that we assume takes place prior to vaccination (see main text). Error bars represent the standard deviation of two

independent simulations of each vaccination protocol (except for the KR-KR-KR and HQ-EU-WT protocols, for which only one successful trial could be obtained), consisting of between one and three immunizations/GC reactions.

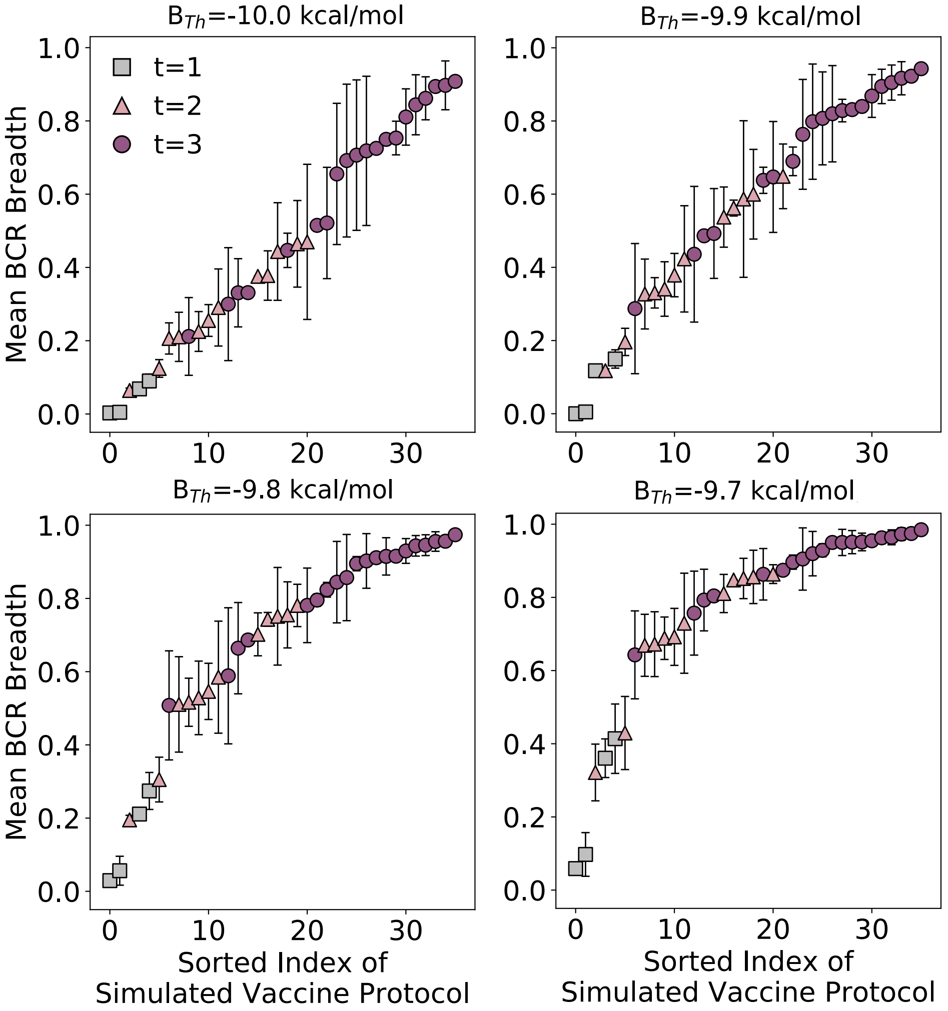

Fig E. Effect of changing the breath threshold (B_{TH}) on the distribution in breadth of the BCRs produced from AM simulations of various vaccination protocols. Mean BCR breadth of vaccination protocols with t=1, 2, and 3 single-Ag sequential immunizations for a breadth threshold of -10.0 kcal/mol (top left), -9.9 kcal/mol (top right; value used in AM simulations), -9.8 kcal/mol (bottom left), and -9.7 kcal/mol (bottom right). Error bars represent the standard deviation of two independent simulations of each vaccination protocol (except for the KR-KR-KR and HQ-EU-WT protocols, for which only one successful trial could be obtained), consisting of between one and three immunizations/GC reactions.

Modeling the B cell receptor/antigen binding free energy

The objective is to find a protocol to compute binding affinities between a given arbitrary sequence of an antibody and an HIV antigen that would require the minimum computer time. At each cycle of the affinity maturation simulation this protocol will be called hundreds of times, so it is imperative to reduce the computational time as much as possible. The methods we use are based on all-atoms descriptions of the antibody-antigen protein-protein complex, so the first task is to generate a reasonable model of the complex. We found that Modeller [8] is the quickest and most reliable method to produce such models, given that an initial template of a related system is available. As templates we use crystal structures of HIV antibodies bound to the gp120 HIV surface protein. The first step of our protocol is thus to create one model of the full complex with Modeller, by grafting the target sequence onto the template. We chose to use a very quick refinement protocol in order to reduce the computation time. Hydrogen atoms are not included in this step, since it was found that in this quick protocol, they often generate the wrong chirality, so only the positions of heavy atoms are generated.

The second step is to optimize the structure. We need to add hydrogen atoms, check for the presence of disulfide bonds, and check for the presence of errors in chirality, in particular around the alpha carbon of each protein residue. Last, the system is energy minimized with 10 steps of steepest descent (SD), followed by 10 steps of adapted-based Newton-Raphson (ABNR). This is expected to fix all mayor problems in the structure, in particular to remove atomic clashes. These checks were all performed using CHARMM [9]. Then the model of the complex is split, to create a model of the ligand (the antibody) and the receptor (the antigen). To compute the binding affinity we use the Rykunov-Fiser statistical pair potentials (RFSPP) [10]. These potentials compute the stability of each protein (G_x), and the binding affinity (dG) can be computed as the difference $dG = G_{cpx} - G_{lig} - G_{rec}$. This whole procedure was repeated 12 times starting with the creation of the model with Modeller using different initial random seeds. The final RFSPP scoring was obtained as the average over the 12 values. Two RFSPP are available: an all-heavy atoms model (RF_HA_SRS) and a beta-carbon directional potential (RF_CB_SRS_OD). While the second is slightly faster, the first produces higher quality results. These scores are not directly usable as binding affinity values and need

to be rescaled to better fit experimental data. The described protocol requires about 12 minutes of computer time to compute one binding affinity (about 1 minute per model), but the models can be run in parallel, requiring only 1 minute of user time on a 12-core machine.

To validate the accuracy of the RFSPP scoring, the computed values were compared with 106 experimental binding affinities of HIV antibody/antigens complexes from the literature [11]. Data were collected for four complexes: the resurfaced stabilized core 3 (RSC3) antigen bound to antibodies VRC01, VRC03, and VRC-PG04, and antigen 93TH057 bound to antibody VRC01. For these four complexes experimental data are available for simple single point mutations and, in the last case, also for few insertions and deletions. For each available experimental dG, the RFSPP score was evaluated as described, and the obtained scores were compared with the experimental dG. As templates we used PDBs 3NGB [12], 3SE8 [13], 3SE9 [13], and 5FYJ [1], respectively, for the four base complexes. The scale and unit of measure of the RFSPP score is uncertain, so a linear regression is performed to find the best parameters (m and q) to interpret the scores as binding affinities in kcal/mol: dGscore = m * RFSPP + q. The whole set of experimental binding affinities was split in half randomly: the first half was used to fit m and q by the linear regression and the second half was used to evaluate the fit, specifically the Pearson (Rp) and Spearman (Rs) correlation coefficients, the Mean Absolute Error (MAE) and Root Mean Square Error (RMSE). Due to the use of a random split in the training and validation sets, the training can be repeated a number of times and different models can be obtained.

To reduce this randomness and avoid picking an arbitrary initial random seed, the full training was repeated 10000 times and all Rp, Rs, MAE and RMSE were stored. To remove all solutions with obviously bad properties (like very low Rp, or very high MAE) a Pareto optimization strategy [14] was adopted. It selects only the models for which no other model exist that have both higher Rp and lower MAE. This procedure constructs a Pareto front that contains only 4 models. Sorting the models in the Pareto front, the first and the last are the ones that optimize the Rp and MAE, respectively. Seeking a model that is balanced between the two limits, the third model of the front was chosen. The obtained linear fit with the RF_HA_SRS statistical pair potential shows a Rp=0.74 (p-value of 1e-9) and Rs=0.74, while the MAE and RMSE are 0.50 kcal/mol and 0.59 kcal/mol, respectively. Fig F shows the

obtained regression. The main negative feature is the low slope of the linear regression, which indicates that the computed dG will be compressed in a smaller range of values with respect to the expected experimental values. The final linear regression result is $dG_{score} = m * RFSPP + q$, with m=0.00619 and q=-8.174. A survey of similar parameters showed qualitatively very similar results.

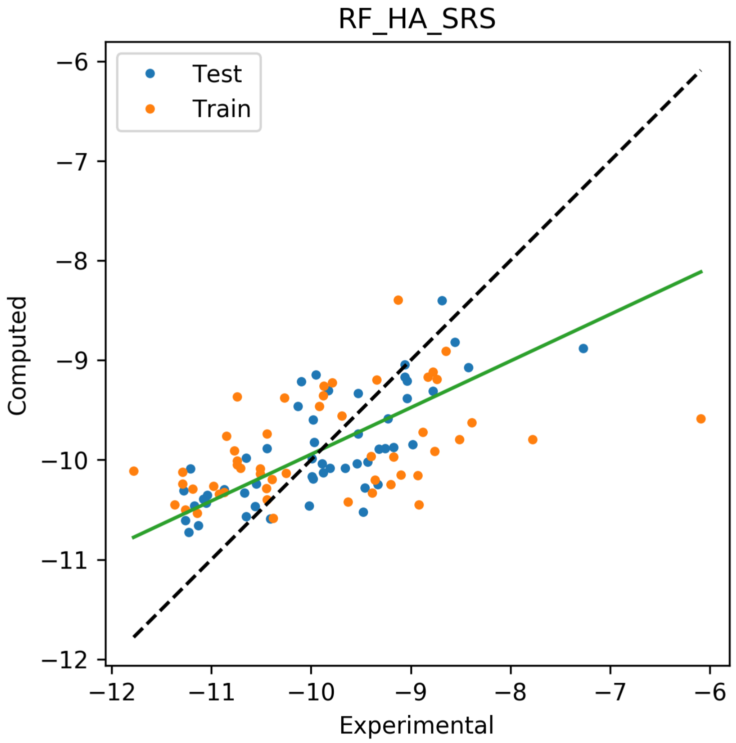

Fig F. Linear correlation between experimental and computed binding affinities using the RF_HA_SRS statistical pair potential. Each dot represents one binding affinity value in kcal/mol. The data in orange are used for the training of the linear regression, while in blue for the validation.

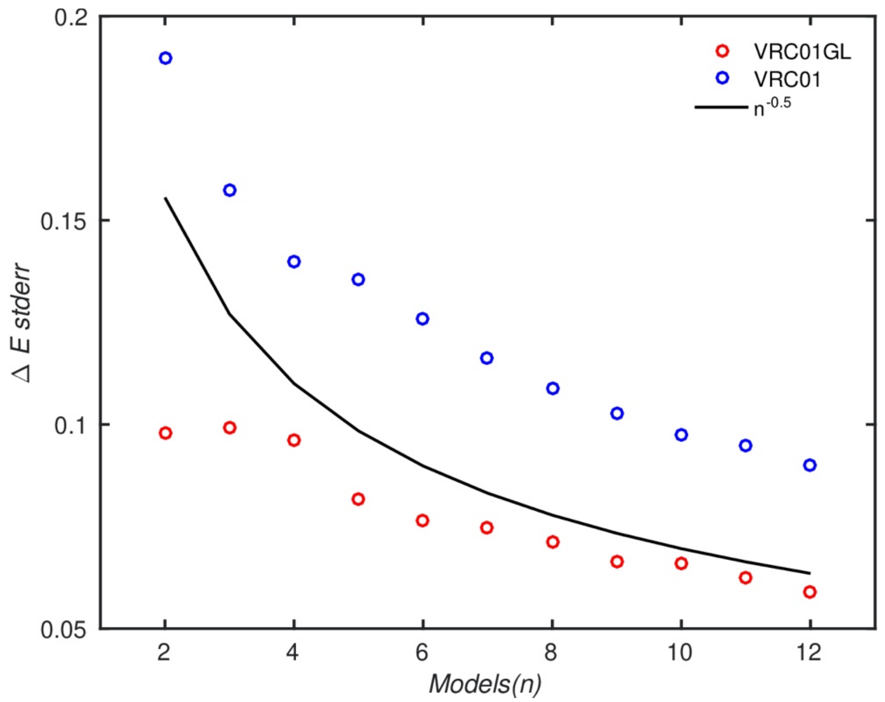

Fig G. Standard error in the computed binding affinity values as a function of the number of structural models for the VRC01 and VRC01GL antibodies bound to the wild-type (WT) antigen.

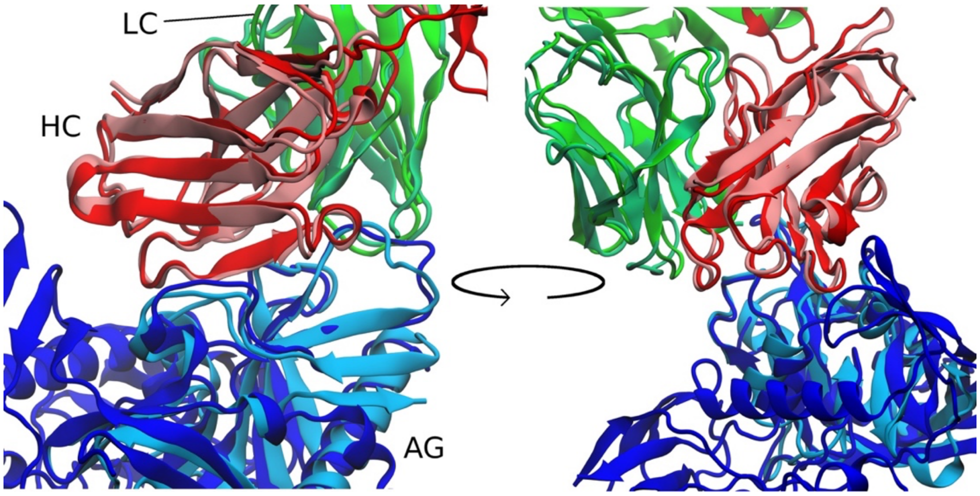

Fig H. Comparison between the binding pose of VRC01 and its germline VRC01GL from experimental structures (PDB: 3NGB and 4JPK, respectively). In blue and cyan is the antigen (AG), in red and pink are the antibody heavy chains (HC), and in lime and green are the antibody light chains (LC), for VRC01 and VRC01GL, respectively.

Scoring function validation: scan for spurious mutations

The affinity maturation protocol involves the stepwise accumulation of mutations from the germline to move towards some mature-like antibody. For the particular case we study here of the germline form of VRC01 evolving against the CD4bs of HIV, we expect that a single point mutation is very unlikely to produce large increases in binding affinity. This expectation stems from the observation that many mutations were required for the germline form of VRC01 to mature into its bnAb form, since the germline Ab can bind only very weakly to native HIV. We also assume that there are no particularly good mutations that always increase the binding affinity. Because of the simplicity of the scoring function, we qualitatively examined its predictions for signs of artifacts or systematic biases; first, we checked that single residue mutations did not cause a large number of very high binding affinity changes. For these reasons we identified 25 residues around the binding site of the VRC01 antibody bound to the BG505 SOSIP antigen and we mutated each of those residues from the germline sequence into each possible amino acid (19 possibilities, excluding the original one), and computed their binding affinity to three antigens: BG505 and the antigens CH115_12 and MW965_26 from the Seaman panel. Fig I, subplot A, shows the range of all 475 (= 25*19) relative change in binding affinity, sorted by magnitude for the three antigens. The highest favorable and unfavorable changes are of only ±2kcal/mol, with the vast majority of mutations being neutral (close to zero). This is the expected behavior for single point random mutations. With the same data, it is also possible to check if some mutations (e.g., mutation to cysteine) are always beneficial mutations. This would imply that a bias towards that particular amino acid is present in the scoring function. The bar plot in Fig I, subplot B, shows the probability that mutating to each amino acid results in an improved binding affinity (i.e., an amino acid with a probability of one means that while scanning the 25 residues in the binding site, mutating any of those resides to that amino acid results in a better binding). Intuitively, we expect that no "preferred" amino acid should exist, which means that the probability should never be close to one, but "terrible" amino acids, with probability close to zero, could exist, e.g., charged amino acids, which are much more sensitive to their environment. The results obtained from antigen BG505 are in good agreement with what is expected, with no preferred mutations (max probability is about 0.6 for W, Y, and H which are all aromatic)

and there are few "terrible" amino acids (D, E, K, and Q, all charged or polar). Antigens CH115_12 and MW965_26 behave similarly.

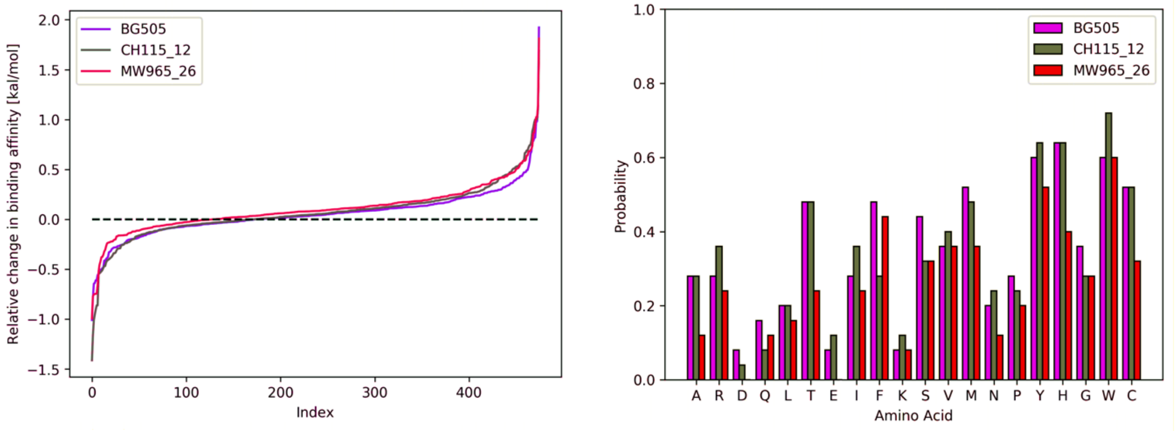

Fig I. A) Relative binding affinity changes while mutating the 25 residues of VRC01GL at the binding site into each possible amino acid, sorted by magnitude. The range of the magnitudes is as expected limited with most mutations neutral (close to zero). B) Bar plot of the probability that mutating to a given amino acid improves the binding affinity. There are no preferred amino acids with very high percentages.

References

1. Stewart-Jones GBE, Soto C, Lemmin T, Chuang GY, Druz A, Kong R, et al. Trimeric HIV-1-Env structures define glycan shields from clades A, B, and G. Cell. 2016 May 5;165: 813–826.
2. Jardine J, Julien J-P, Menis S, Ota T, Kalyuzhniy O, McGuire A, et al. Rational HIV immunogen design to target specific germline B cell receptors. Science. 2013 May 10;340: 711–716.
3. Conti S, Kaczorowski KJ, Song G, Porter K, Andrabi R, Burton DR, et al. Design of immunogens to elicit broadly neutralizing antibodies against HIV targeting the CD4 binding site. Proc Natl Acad Sci. 2021 Mar 2;118: e2018338118.
4. Humphrey, William, Dalke, Andrew, Schulten K. VMD: Visual molecular dynamics. J Mol Graph. 1996 Feb;14: 33–38.

5. Kwong PD, Mascola JR, Nabel GJ. Broadly neutralizing antibodies and the search for an HIV-1 vaccine: The end of the beginning. Nat Rev Immunol. 2013;13: 693–701.

6. Wang S, Mata-Fink J, Kriegsman B, Hanson M, Irvine DJ, Eisen HN, et al. Manipulating the selection forces during affinity maturation to generate cross-reactive HIV antibodies. Cell. 2015 Feb 12;160: 785–797.

7. Yaari G, Vander Heiden JA, Uduman M, Gadala-Maria D, Gupta N, Joel JN, et al. Models of somatic hypermutation targeting and substitution based on synonymous mutations from high-throughput immunoglobulin sequencing data. Front Immunol. 2013;4: 1–11.

8. Webb B, Sali A. Comparative protein structure modeling using MODELLER. Curr Protoc Bioinforma. 2016;54: 5.6.1-5.6.37.

9. Brooks BR, Bruccoleri RE, Olafson BD, States DJ, Swaminathan S, Karplus M. CHARMM: A program for macromolecular energy, minimization, and dynamics calculations. J Comput Chem. 1983;4: 187–217.

10. Rykunov D, Fiser A. New statistical potential for quality assessment of protein models and a survey of energy functions. BMC Bioinformatics. 2010 Mar 12;11: 128.

11. Seaman MS, Janes H, Hawkins N, Grandpre LE, Devoy C, Giri A, et al. Tiered categorization of a diverse panel of HIV-1 Env pseudoviruses for assessment of neutralizing antibodies. J Virol. 2010 Feb 1;84: 1439–1452.

12. Zhou T, Georgiev I, Wu X, Yang ZY, Dai K, Finzi A, et al. Structural basis for broad and potent neutralization of HIV-1 by antibody VRC01. Science. 2010 Aug 13;329: 811–817.

13. Wu X, Zhou T, Zhu J, Zhang B, Georgiev I, Wang C, et al. Focused evolution of HIV-1 neutralizing antibodies revealed by structures and deep sequencing. Science. 2011 Sep 16;333: 1593–1602.

14. Jahan A, Edwards KL. Multi-Criteria Decision Analysis for Supporting the Selection of Engineering Materials in Product Design. 2013. doi:10.1016/C2012-0-02834-7.
